# State-of-the-Art Deep Learning Methods on Electrocardiogram Data: Systematic Review

**DOI:** 10.2196/38454

**Published:** 2022-08-15

**Authors:** Georgios Petmezas, Leandros Stefanopoulos, Vassilis Kilintzis, Andreas Tzavelis, John A Rogers, Aggelos K Katsaggelos, Nicos Maglaveras

**Affiliations:** 1 Lab of Computing, Medical Informatics and Biomedical-Imaging Technologies The Medical School Aristotle University of Thessaloniki Thessaloniki Greece; 2 Department of Biomedical Engineering Northwestern University Evanston, IL United States; 3 Department of Material Science Northwestern University Evanston, IL United States; 4 Department of Electrical and Computer Engineering Northwestern University Evanston, IL United States

**Keywords:** electrocardiogram, ECG, ECG databases, deep learning, convolutional neural networks, CNN, residual neural network, ResNet, long short-term memory, LSTM, diagnostic tools, decision support, clinical decision

## Abstract

**Background:**

Electrocardiogram (ECG) is one of the most common noninvasive diagnostic tools that can provide useful information regarding a patient’s health status. Deep learning (DL) is an area of intense exploration that leads the way in most attempts to create powerful diagnostic models based on physiological signals.

**Objective:**

This study aimed to provide a systematic review of DL methods applied to ECG data for various clinical applications.

**Methods:**

The PubMed search engine was systematically searched by combining “deep learning” and keywords such as “ecg,” “ekg,” “electrocardiogram,” “electrocardiography,” and “electrocardiology.” Irrelevant articles were excluded from the study after screening titles and abstracts, and the remaining articles were further reviewed. The reasons for article exclusion were manuscripts written in any language other than English, absence of ECG data or DL methods involved in the study, and absence of a quantitative evaluation of the proposed approaches.

**Results:**

We identified 230 relevant articles published between January 2020 and December 2021 and grouped them into 6 distinct medical applications, namely, blood pressure estimation, cardiovascular disease diagnosis, ECG analysis, biometric recognition, sleep analysis, and other clinical analyses. We provide a complete account of the state-of-the-art DL strategies per the field of application, as well as major ECG data sources. We also present open research problems, such as the lack of attempts to address the issue of blood pressure variability in training data sets, and point out potential gaps in the design and implementation of DL models.

**Conclusions:**

We expect that this review will provide insights into state-of-the-art DL methods applied to ECG data and point to future directions for research on DL to create robust models that can assist medical experts in clinical decision-making.

## Introduction

### Study Background

Electrocardiogram (ECG) is one of the most common noninvasive diagnostic tools used in clinical medicine [1]. An ECG is a nonstationary physiological signal that measures voltage changes produced by the electrical activity of the heart. It is mostly used by cardiologists to assess heart function and electrophysiology [2]. ECG interpretation plays a vital role in personalized medicine and can assist in cardiovascular disease (CVD) detection, rehabilitation, and the development of treatment strategies. Owing to the major increase in the amount of ECG data available and measurement heterogeneity from medical devices and placements, there are many cases where traditional diagnosis becomes inefficient, as it requires complex manual analysis and highly trained medical experts to achieve adequate accuracy [3].

During the past few decades, the massive surge in computational power and availability of large data sets have created new opportunities for machine-driven diagnosis in many health care areas [4]. Artificial intelligence (AI) is leading the way in most attempts to develop reliable diagnostic tools based on data-driven techniques [5]. In particular, deep learning (DL) algorithms, a subset of machine learning (ML), can generate powerful models that can learn relationships between data and reveal hidden patterns in complex biomedical data without the need for prior knowledge. DL models adjust better to large data sets and, in most cases, continue to improve with the addition of more data, thus enabling them to outperform most classical ML approaches [6,7]. They have been tested extensively in many application areas, such as speech recognition, visual object recognition, object detection, and natural language processing, achieving promising results [8].

DL algorithms are typically based on deep network architectures comprising multiple hidden layers [9]. The most frequently used DL algorithms are convolutional neural networks (CNNs), which were originally proposed for object recognition and image classification [10,11]. Since then, they have been successfully used in various medical applications, including medical image analysis [12], biomedical signal classification [13,14], pulmonary sound classification [15], biomedical signal quality assessment [16], pathological voice detection [17], and sleep staging [18].

Moreover, residual neural networks (ResNets) [19], which were recently proposed to solve the difficulties of training very deep neural networks (DNNs), are well established and used in several medical tasks, such as prostate cancer detection [20], nuclei segmentation and detection [21], coronary calcium detection [22], and pulmonary nodule classification [23].

In addition to CNN and ResNet architectures, recurrent neural networks (RNNs) represent another type of DL technique frequently used in health care. Disease prediction [24], biomedical image segmentation [25], and obstructive sleep apnea detection [26] are only a few of their applications. More specifically, the performance of improved versions of classic RNNs, such as long short-term memory (LSTM) networks and gated recurrent units (GRUs), has been studied extensively in recent years in a series of health-related tasks, including medical image denoising [27], Alzheimer disease detection [28], life expectancy prediction [29], cardiac arrhythmia classification [30], epileptic seizure detection [31], cell segmentation [32], and cardiac phase detection [33].

Another DL method proposed in 2017 that has recently gained popularity among the scientific community is transformers [34], which adopts the mechanism of self-attention to handle sequential data. They have been tested in a series of medical tasks, including cardiac abnormality diagnosis [35], food allergen identification [36], medical language understanding [37], and chemical image recognition [38].

Finally, autoencoders, a DNN technique capable of learning compressed representations of its inputs, have been tested in several medical applications, such as the prediction of heart transplant rejection [39], cell detection and classification [40], anticancer drug response classification [41], premature ventricular contraction detection [42], and endomicroscopic image classification [43].

The purpose of this study is to provide a complete and systematic account of the current state-of-the-art DL methods for ECG data. The main idea behind this comprehensive review is to group and summarize the DL approaches per field of application, discuss the most notable studies, and provide a detailed overview of the major ECG databases. In addition, we will identify important open research problems and directions and provide an assessment of the future of the field. We expect this review to be of great value to newcomers to the topic, as well as to practitioners in the field.

The remainder of this paper is structured as follows: In the *Background of DL* section, background knowledge for DL techniques and algorithms is presented, and related state-of-the-art methods for ECG processing and analysis are reviewed. In the *Methods* section, the research methodology is described in detail, and, in the *Results* section, the results of the systematic review are presented. In the *Discussion* section, a discussion based on the research findings is presented. Finally, the conclusions of the study are summarized in the *Conclusions* section.

### Background of DL

#### DL Algorithm

DL is a branch of ML that uses multilayered structures of algorithms called neural networks (NNs) to learn representations of data by using multiple levels of abstraction [8]. Unlike most traditional ML algorithms, many of which have a finite capacity to learn regardless of how much data they acquire, DL systems can usually improve their performance with access to more data.

Given the availability of large data sets and advancements in modern technology, DL has seen a spectacular rise in the past decade. DL algorithms can construct robust data-driven models that can reveal hidden patterns in data and make predictions based on them. The following subsections describe some of the most commonly used DL methods that are applied to a wide range of health-related tasks where ECG data are present.

#### CNN Algorithm

CNNs are among the most popular DL architectures and owe their name to the mathematical concept of convolution. CNNs are designed to adaptively learn the spatial hierarchy of data by extracting and memorizing high- and low-level patterns to predict the final output.

Although they were initially designed to deal with 2D image data [44], during the past few years, several modified 1D versions of them have been proposed for numerous applications, achieving state-of-the-art performance [45].

The structure of a typical CNN integrates a pipeline of multiple hidden layers, in particular, convolutional and pooling layers, followed by fully connected layers. The convolutional layers implement filters (or kernels) that perform convolution between the kernel (impulse response of the filter) and the input signal. In this way, each convolutional layer creates features (or activation maps) from its input, a process commonly known as feature extraction.

In contrast, the pooling layers conduct down-sampling of the extracted feature maps to reduce the computational complexity required when processing large volumes of data. Finally, the fully connected layers are simple feed-forward NNs that create weighted connections between successive layers. Therefore, they achieve the mapping of the aggregated activations of all previous layers into a class probability distribution by applying a sigmoid or *softmax* activation function that represents the final output of the CNN.

#### ResNet Algorithm

ResNet is a special type of DL network that was proposed to solve the vanishing gradient problem, which occurs when training DNNs. In other words, as the number of stacked layers of a DNN increases, the gradient of the earlier layers vanishes. Thus, the network fails to update the weights of the earlier layers. This means that no learning occurs in the earlier layers, resulting in poor training and testing performance.

The key idea behind ResNet is the introduction of residual blocks that use skip connections to add the outputs from earlier layers to those of later layers. Precisely, the network creates shortcuts that enable the gradient to take shorter paths through the deeper layers, thereby eliminating the vanishing gradient problem. Thus, the precision of deep feature extraction is improved, whereas the computational complexity of the network remains substantially low.

ResNet is typically a network comprising CNN blocks that are successively repeated multiple times. Many variants of the ResNet architecture use the same concept but various numbers of layers to address different problems, such as ResNet-34, ResNet-50, and ResNet-101, where 34, 50, and 101 are the depths of the network, respectively.

#### RNN Algorithm

RNNs were first introduced by Rumelhart et al [46] in 1986. They are a class of artificial NNs capable of memorizing the temporal dynamics of sequential data by forming a directed graph along them. Specifically, they deploy hidden units that create strong dependencies among data by preserving valuable information regarding previous inputs to predict current and future outputs.

However, as the time distance between dependent inputs increases, RNNs become incapable of handling long-term dependencies because of the vanishing gradient problem. To address this problem, new variations of RNNs have been proposed, including LSTM networks and GRUs.

LSTM networks were introduced by Hochreiter and Schmidhuber [47] in 1997. They solved the problem of long-term dependencies by implementing gates to control the memorization process. This means that they can recognize and retain both the long- and short-term dependencies between the data of a sequential input for long periods, resulting in efficient learning and, finally, improved performance.

The structure of LSTM comprises an ordered chain of identical cells. Each cell is responsible for transferring 2 states to the next cell, namely, the current internal cell state and its internal hidden state, also known as short-term and long-term memory, respectively. To achieve this, it uses 3 types of gates, namely forget, input, and output gates, to control the information that is passed onto further computations.

Specifically, using the forget gate, the cell determines which part of the previous time stamp’s information needs to be retained and which should be forgotten. The input gate updates the cell state by adding new information. Finally, the output gate selects information that will be passed on as the output of the cell. By controlling the process of adding valuable information or removing unnecessary information, a cell can remember long-term dependencies over arbitrary time intervals.

In contrast, motivated by the LSTM unit, in 2014, Cho et al [48] proposed GRUs to address the vanishing gradient problem. Unlike LSTMs, GRUs do not have separate cell states. In addition, they use only 2 gates to control the flow of information via the hidden state, namely, the update and reset gates.

Precisely, the update gate, which acts as the unit’s long-term memory, is responsible for selecting the amount of previous information that must be passed on to the current hidden state. By contrast, the reset gate represents the short-term memory of the unit and oversees the determination of the amount of past information that must be ignored.

With these 2 gates, each hidden unit can capture dependencies over different time scales. Thus, units trained to capture long-term dependencies tend to have update gates that are mostly active, and conversely, those trained to memorize short-term dependencies tend to have active reset gates.

#### Autoencoders

Autoencoders are a special type of feed-forward NNs that was introduced by Rumelhart et al [49] in 1986. An autoencoder can learn efficient representations of data and is mainly applied for feature extraction and dimensionality reduction.

A typical autoencoder structure includes 2 parts: encoder and decoder. The encoder compresses the input and creates a latent representation, which is mapped to a hidden layer, also known as a bottleneck. Then, the decoder uses this latent representation to reconstruct the original input.

In this manner, an autoencoder is trained by minimizing the reconstruction error to learn to create low-dimensional copies of higher-dimensional data. There are several types of autoencoders, including denoising autoencoders [50], variational autoencoders [51], and convolutional autoencoders [52].

## Methods

### Literature Search

**Figure 1:**
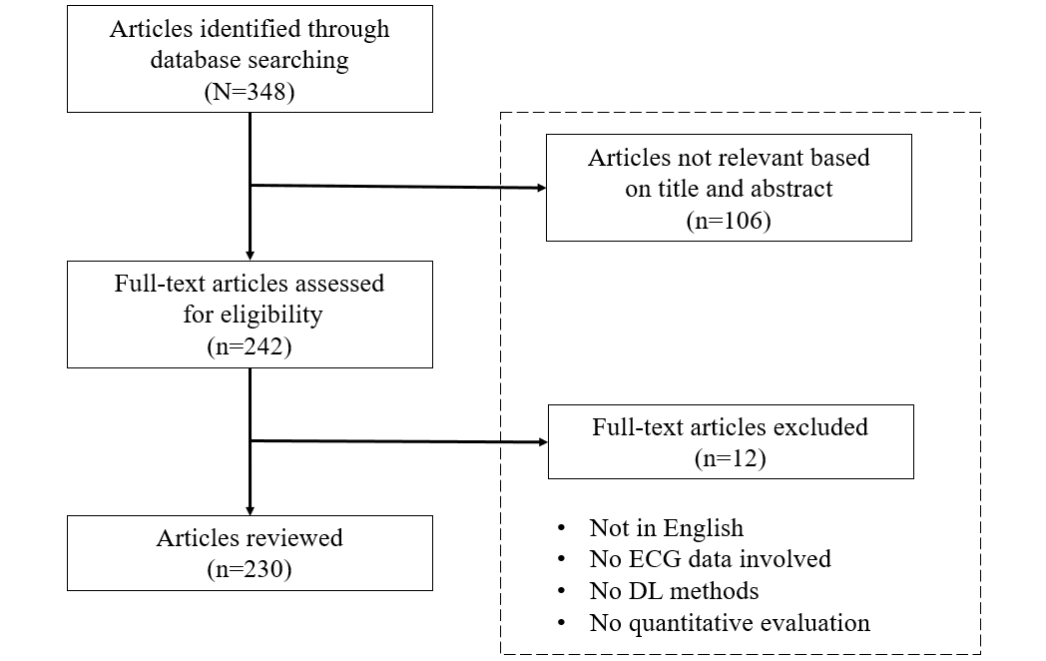
Flow diagram of the literature search. DL: deep learning; ECG: electrocardiogram.

The PubMed search engine was systematically searched by combining “deep learning” and keywords such as “ecg,” “ekg,” “electrocardiogram,” “electrocardiography,” and “electrocardiology.” During the initial screening, 348 unique articles published in various journals between January 2020 and December 2021 were identified. Of these 348 articles, 106 (30.5%) were excluded based on their titles and abstracts, and the remaining 242 (69.5%) were further reviewed. The reasons for article exclusion were manuscript written in any language other than English, absence of ECG data or DL methods involved in the study, and absence of a quantitative evaluation of the proposed approaches. After a full-text assessment, 4.9% (12/242) of the articles were excluded as they were about works that did not include ECG signals. Finally, 230 relevant articles were selected for this review. The detailed process of the literature search and selection is illustrated in Figure 1.

### Bibliometric Analysis

**Figure 2:**
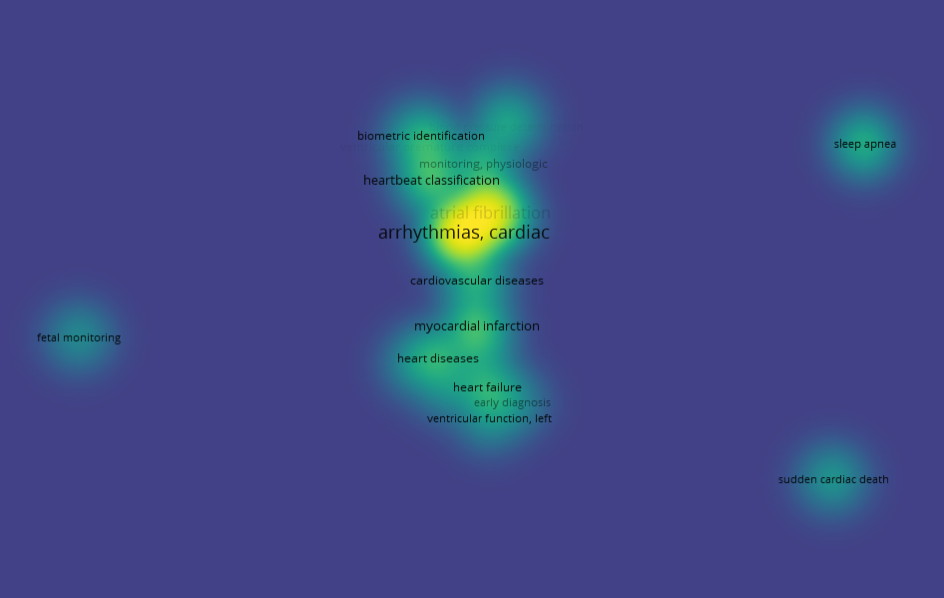
The co-occurrence network for the “clinical issues” cluster.

**Figure 3:**
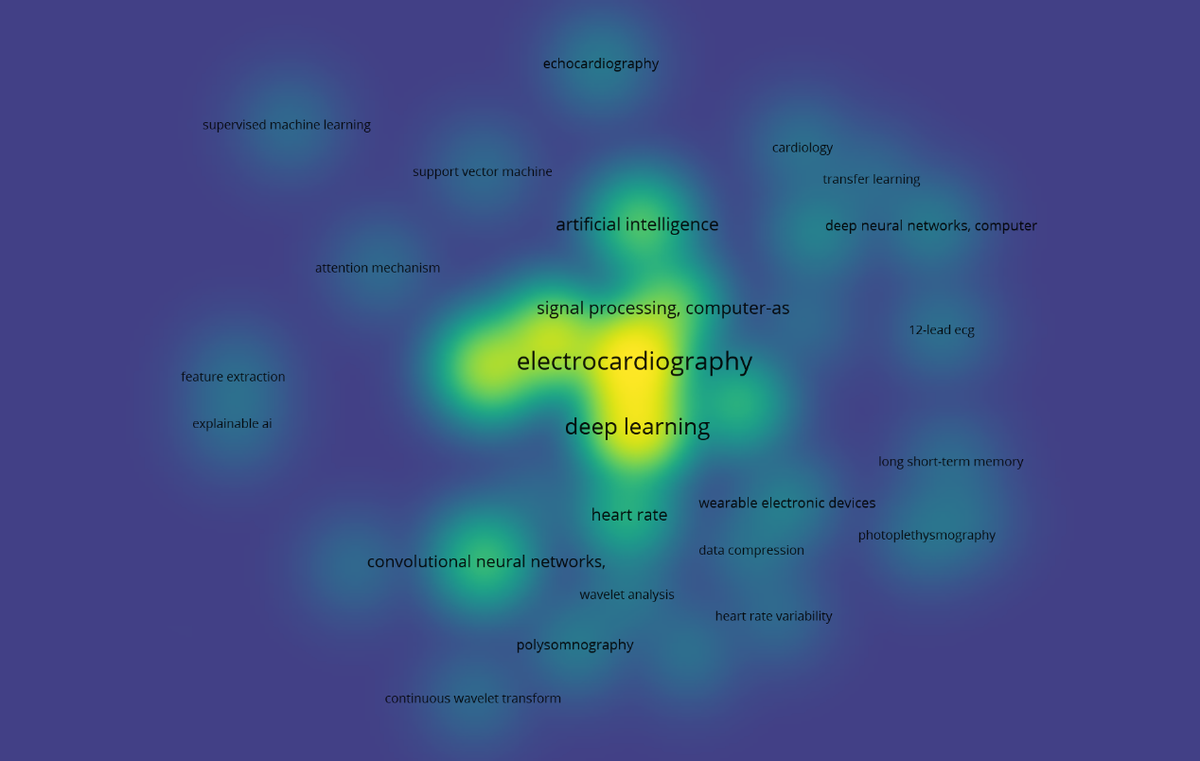
The co-occurrence network for the “methods and tools” cluster.

**Figure 4:**
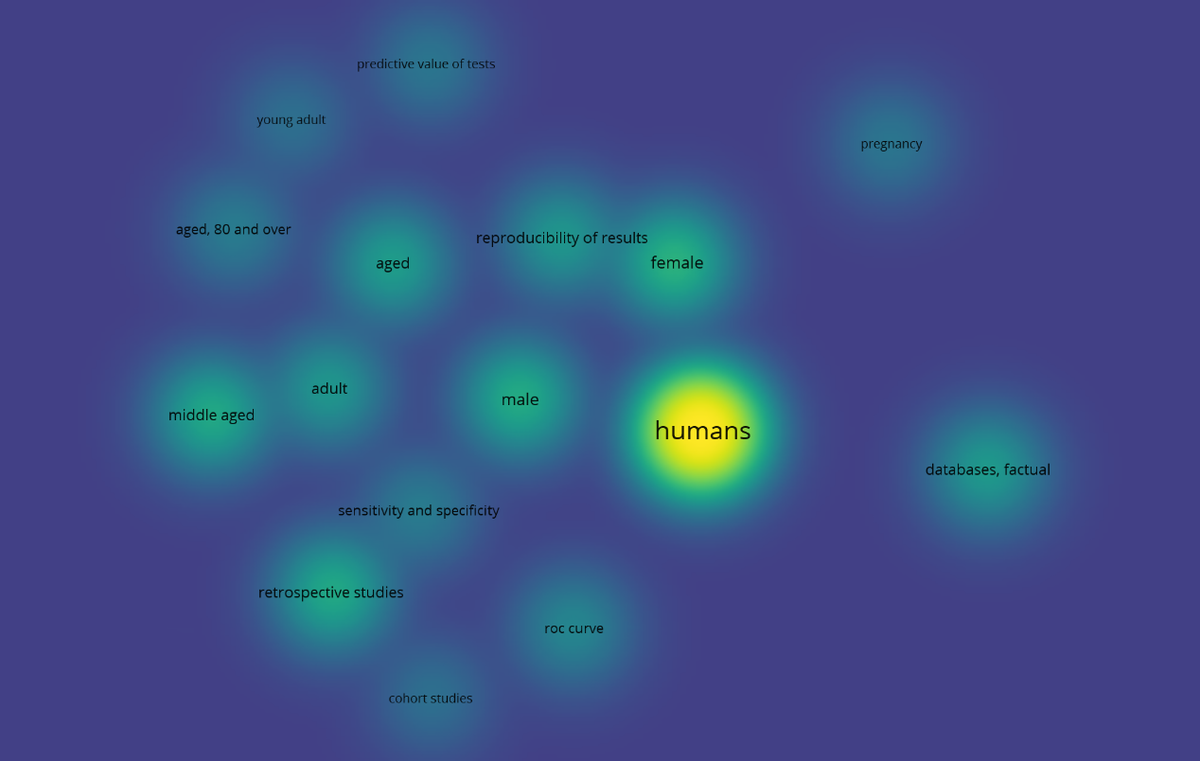
The co-occurrence network for the “study characteristics” cluster.

To obtain a clear picture of the literature search results, a co-occurrence analysis was conducted. For this purpose, the VOSviewer software tool (Nees Jan van Eck and Ludo Waltman) [53] was used to create and visualize 3 maps based on the bibliographic data of this study. Specifically, all keywords from the 230 relevant studies were grouped and linked to establish the impact of each keyword on the given scientific field and its interconnections with other keywords. In this way, 3 distinct clusters of keywords were formed, namely “clinical issues” (cluster 1), “methods and tools” (cluster 2), and “study characteristics” (cluster 3), as shown in Textbox 1, and an individual map was generated for each of the 3 categories. Figure 2 displays the co-occurrence network that corresponds to the “clinical issues” cluster of keywords. Cardiac arrhythmias and atrial fibrillation (AF) were identified as the major clinical issues in this review. Figure 3 presents the co-occurrence network for the “methods and tools” cluster, where ECG and DL constitute the network’s core. Finally, Figure 4 shows the co-occurrence network for the “study characteristics” cluster, where, as expected, humans are the center of attention.

**Keyword cluster summary.**

**Cluster and keywords**

- Cluster 1
  “arrhythmias, cardiac,” “atrial fibrillation,” “biometric identification,” “blood pressure determination,” “cardiomyopathy,” “cardiovascular diseases,” “coronary artery disease,” “covid-19,” “early diagnosis,” “fetal monitoring,” “heart diseases,” “heart failure,” “heartbeat classification,” “hypertension,” “monitoring, physiologic,” “myocardial infarction,” “sleep apnea,” “sudden cardiac death,” “ventricular fibrillation,” “ventricular function, left,” “ventricular premature complexes”
- Cluster 2
  “12-lead ecg,” “algorithms,” “artificial intelligence,” “attention mechanism,” “blood pressure,” “cardiology,” “continuous wavelet transform,” “convolutional neural networks, computer,” “data compression,” “deep learning,” “deep neural networks, computer,” “diagnosis, computer-assisted,” “echocardiography,” “electrocardiography,” “electroencephalography,” “feature extraction,” “feature fusion,” “heart,” “heart rate,” “heart rate variability,” “long short-term memory,” “machine learning,” “neural networks, computer,” “photoplethysmography,” “polysomnography,” “recurrent neural networks, computer,” “signal processing, computer-assisted,” “supervised machine learning,” “support vector machine,” “wavelet analysis,” “wearable electronic devices”
- Cluster 3
  “adult,” “aged,” “aged, 80 and over,” “cohort studies,” “databases, factual,” “female,” “humans,” “male,” “middle aged,” “predictive value of tests,” “pregnancy,” “reproducibility of results,” “retrospective studies,” “roc curve,” “sensitivity and specificity,” “young adult”

## Results

### ECG Data Sources

On the basis of the selected studies, multiple ECG data sources were identified, including several well-established publicly available databases. These data sources exhibit differences in the number of enrolled patients, number of recordings, ECG systems used to collect them, data duration, and sample rate. Their content is presented in Multimedia Appendix 1 [54-92], where the links to publicly available data are placed as hyperlinks on the name of each database.

The most commonly used databases were the Massachusetts Institute of Technology (MIT)–Beth Israel Hospital (BIH) Arrhythmia Database [80] (55/230, 23.9% studies), 2017 PhysioNet/CinC Challenge database [57] (31/230, 13.5% studies), the China Physiological Signal Challenge (CPSC) 2018 database [69] (26/230, 11.3% studies), the MIT-BIH Atrial Fibrillation Database [81] (17/230, 7.4% studies), and the *Physikalisch Technische Bundesanstalt* (PTB)–XL ECG data set [87] (17/230, 7.4% studies).

The MIT-BIH Arrhythmia Database contains 48 half-hour excerpts of 2-channel ambulatory ECG recordings obtained from 47 participants studied by the BIH Arrhythmia Laboratory between 1975 and 1979 with a sampling frequency of 360 Hz. Of these, 23 recordings were chosen at random from a set of 4000 recordings of 24-hour ambulatory ECG collected from a mixed population of inpatients (approximately 60%) and outpatients (approximately 40%) at Boston’s BIH, whereas the remaining 25 recordings were selected from the same set to include less common but clinically significant arrhythmias that would not be well represented in a small random sample. Finally, each recording was independently annotated by ≥2 cardiologists.

In contrast, the 2017 PhysioNet/CinC Challenge database contains 12,186 single-lead ECG recordings collected using a sampling frequency of 300 Hz. The training set contains 8528 single-lead ECG recordings lasting from 9 seconds to just >60 seconds, and the test set contains 3658 ECG recordings of similar lengths.

The CPSC 2018 database comprises ECG recordings collected from 11 hospitals by using a sampling frequency of 500 Hz. The training set contains 6877 (female: 3178; male: 3699) 12-lead ECG recordings lasting from 6 seconds to 60 seconds, and the test set, which is unavailable to the public for scoring purposes, contains 2954 ECG recordings of similar lengths.

Furthermore, the MIT-BIH Atrial Fibrillation Database includes 25 long-term ECG recordings of human patients with AF (mostly paroxysmal). The individual recordings are each 10 hours in duration and contain 2 ECG signals, each sampled at 250 Hz, whereas the rhythm annotation files were manually prepared and contain rhythm annotations of 4 types, namely, AFIB (AF), AFL (atrial flutter), J (AV junctional rhythm), and N (all other rhythms).

Finally, the PTB-XL ECG data set is a large data set of 21,837 clinical 12-lead ECGs from 18,885 patients with a duration of 10 seconds and a sampling frequency of 500 Hz. The raw waveform data were annotated by up to 2 cardiologists who assigned multiple ECG statements to each record.

### Medical Applications

#### Overview

The 230 relevant articles identified during the literature search were grouped into several categories based on their study objectives. In particular, 6 distinct medical applications were identified: blood pressure (BP) estimation, CVD diagnosis, ECG analysis, biometric recognition, sleep analysis, and other clinical analyses.

Most of the studies use ECG signals for CVD diagnosis, mainly via signal or beat classification. Moreover, a significant portion of them uses DL algorithms to perform ECG analysis, as well as diagnosis of other clinical conditions.

In this study, the identified DL approaches are grouped per field of application, and the most notable approaches are discussed in detail. Moreover, Multimedia Appendix 2 [93-322] provides details regarding the author and the year of publication of each article, the medical task that each article refers to, data, data preprocessing, splitting strategy, DL algorithm applied in each study, and performance of each approach.

#### BP Estimation

Only 2.6% (6/230) of studies that applied DL methods to ECG data to perform BP estimation were identified in the literature search. A combined architecture of ResNets and LSTM was proposed twice (33.3%), once by Miao et al [94], who achieved a mean error of −0.22 (SD 5.82) mm Hg for systolic BP (SBP) prediction and of −0.75 (SD 5.62) mm Hg for diastolic BP (DBP) prediction using data that originated from a private database, and once by Paviglianiti et al [96], who achieved a mean average error of 4.118 mm Hg for SBP and 2.228 mm Hg for DBP prediction using the Medical Information Mart for Intensive Care database. By contrast, Jeong and Lim [98] exercised a CNN-LSTM network on the Medical Information Mart for Intensive Care database and managed to predict SBP and DBP with a mean error of 0.0 (SD 1.6) mm Hg and 0.2 (SD 1.3) mm Hg, respectively.

#### CVD Diagnosis

More than half (152/230, 66.1%) of the studies that were identified during the literature search applied DL methods to ECG data for CVD diagnosis. The most common data sources for CVD diagnosis are private (37%) and mixed public (25%) databases. However, a notable proportion (15%) of the aforementioned studies exclusively used the MIT-BIH Arrhythmia Database. Almost the half of them (10/23, 43.5%) applied a CNN structure.

Regarding the MIT-BIH Arrhythmia Database, the best accuracy (99.94%) was achieved by Wang et al [185], who introduced a fused autoencoder-CNN network to classify 6 different ECG rhythms. However, a high percentage of the studies that managed to classify data originating from the same database implemented a CNN structure. Lu et al [180] used a 1D-CNN for arrhythmia classification, achieving an accuracy of 99.31%, whereas Yu et al [219] used a 1D-CNN to detect premature ventricular contraction, achieving a classification accuracy of 99.70%.

On the contrary, a ResNet architecture was tested only 3 times on the MIT-BIH Arrhythmia Database; nonetheless, 0.9% (2/230) of these studies showed a high model performance. In particular, Li et al [146] proposed a ResNet model for heartbeat classification, achieving a classification accuracy of 99.38%, whereas Zhang et al [211] used a ResNet-101 structure to classify ECG beats with transfer learning and achieved an accuracy of 99.75%.

Regarding the rest of the databases, several noteworthy studies were identified in the literature. Specifically, Cai et al [101] implemented a densely connected DNN on a private ECG database for AF detection, achieving an accuracy between 97.74% and 99.35% for 3 different classification tasks, whereas Ghosh et al [103] applied a hierarchical extreme learning machine to ECG data from multiple public databases, achieving an accuracy of 99.40% in detecting AF.

Furthermore, Butun et al [125] proposed a 1D-capsule NN for the detection of coronary artery disease, achieving classification accuracies of 99.44% and 98.62% on 2-second and 5-second ECG segments, respectively, originating from a private ECG database. Another study by Thiagarajan et al [129] used multiple convolutional and pooling layers within a structure named DDxNet on ECG data from 2 public databases, achieving an accuracy of 98.50% for arrhythmia classification and 99.90% for myocardial infarction detection.

A study by Radhakrishnan et al [163] evaluated the performance (sensitivity 99.17%, specificity 99.18%, and accuracy 99.18%) of a 2D bidirectional LSTM network to detect AF in ECG signals from 4 public databases, whereas Petmezas et al [170] tested (sensitivity 97.87% and specificity 99.29%) a CNN-LSTM model on ECG signals originating from the MIT-BIH Atrial Fibrillation Database for the same medical task.

Moreover, Jahmunah et al [192] applied a CNN architecture to ECG data from several public ECG databases to detect coronary artery disease, myocardial infarction, and congestive heart failure, achieving an accuracy of 99.55%. Another study by Dai et al [195] proposed a CNN for CVD diagnosis using different intervals of ECG signals from the PTB Diagnostic ECG Database and achieved accuracies of 99.59%, 99.80%, and 99.84% for 1-, 2-, and 3-second ECG segments, respectively.

Finally, Ma et al [208] introduced an improved dilated causal CNN to classify ECG signals from the MIT-BIH Atrial Fibrillation Database, achieving a high model performance (sensitivity 98.79%, specificity 99.04%, and accuracy 98.65%), whereas Zhang et al [238] tested (sensitivity 99.65%, specificity 99.98%, and accuracy 99.84%) a CNN for AF detection on ECG signals from 2 major public databases.

#### ECG Analysis

In total, 12.6% (29/230) of studies that applied DL methods to ECG data to perform ECG analysis were identified during the literature search. Once again, CNN was the most commonly used DL method (11/29, 38%); nonetheless, the best model accuracy was achieved by studies using other DL methods. In particular, Teplitzky et al [251] tested (sensitivity 99.84% and positive predictive value 99.78%) a hybrid approach that combines 2 DL approaches, namely BeatNet and RhythmNet, to annotate ECG signals that originated from both public and private ECG databases, whereas Murat et al [258] used a CNN-LSTM approach on ECG data from the MIT-BIH Arrhythmia Database and achieved an accuracy of 99.26% in detecting 5 types of ECG beats.

By contrast, Vijayarangan et al [261] used a fused CNN-ResNet structure to perform R peak detection in ECG signals from several public ECG databases and achieved *F*_1_-scores between 96.32% and 99.65% for 3 testing data sets. Another study by Jimenez Perez et al [265] implemented a U-Net model to delineate 2-lead ECG signals originating from the QT Database and achieved sensitivities of 98.73%, 99.94%, and 99.88% for P wave, QRS complex, and T wave detection, respectively. Finally, a study by Strodthoff et al [274] used a ResNet for patient sex identification by using 12-lead ECG recordings lasting between 6 and 60 seconds from several public databases and achieved an area under the curve of 0.925 for the PTB-XL ECG data set and 0.974 for the CPSC 2018 database.

#### Biometric Recognition

Only 3% (7/230) of studies that applied DL methods to ECG data to perform biometric recognition were identified in the literature search. Although 57% (4/7) of the studies used a CNN architecture, only 29% (2/7) of them achieved high model performance. Specifically, Wu et al [284] achieved an identification rate of >99% by using ECG signals from 2 public databases, whereas Chiu et al [285] achieved an identification rate of 99.10% by using single-lead ECG recordings that originated from the PTB Diagnostic ECG Database.

On the contrary, Song et al [281] implemented a ResNet-50 architecture for person identification using multiple ECG, face, and fingerprint data from several public and private databases and achieved an accuracy of 98.97% for ID classification and 96.55% for gender classification. Finally, AlDuwaile and Islam [283] tested several pretrained models, including GoogleNet, ResNet, MobileNet, and EfficientNet, and a CNN model to perform human recognition using ECG signals that originated from 2 public databases and achieved an accuracy between 94.18% and 98.20% for ECG-ID mixed-session and multisession data sets.

#### Sleep Analysis

Approximately 5.2% (12/230) of studies that applied DL methods to ECG data to perform sleep analysis were identified during the literature search. Half (6/12, 50%) of the studies proposed a CNN model, some of which achieved high performance in several sleep analysis–related tasks. In particular, Chang et al [289] used 1-minute ECG segments from the Apnea-ECG Database and designed a CNN to detect sleep apnea, achieving an accuracy of 87.90% and 97.10% for per-minute and per-recording classification, respectively.

In addition, a study by Urtnasan et al [291] proposed a CNN for the identification of sleep apnea severity by using ECG segments from a private database and achieved an *F*_1_-score of 98.00%, whereas another study by Urtnasan et al [297] implemented a CNN to classify sleep disorders by using polysomnography recordings from the Cyclic Alternating Pattern Sleep Database and achieved *F*_1_-scores between 95% and 99% for 5 different sleep disorder categories. By contrast, Nasifoglu and Erogul [295] tested a fused CNN-ResNet approach for obstructive sleep apnea detection (accuracy 85.20%) and prediction (accuracy 82.30%) using data from a private database. Mukherjee et al [296] used a multilayer perceptron to detect sleep apnea from ECG recordings that originated from the Apnea-ECG Database, achieving an accuracy of 85.58%.

#### Other Clinical Analyses

Approximately 10.4% (24/230) of studies that applied DL methods to ECG data to perform other clinical analyses were identified during the literature search. Almost half (10/24, 42%) of the studies proposed a CNN approach, including Isasi et al [300], who used data from a private database to detect shockable and nonshockable rhythms during cardiopulmonary resuscitation with an accuracy of 96.10%, and Ozdemir et al [309], who used a private database to diagnose COVID-19 through ECG classification (accuracy 93.00%).

Other notable works include a study by Chang et al [311], which tested (sensitivity 84.60% and specificity 96.60%) an ECG12Net to detect digoxin toxicity by using private ECG signals from patients with digoxin toxicity and patients in the emergency room, and another study by Baghersalimi et al [313], which evaluated the performance (sensitivity 90.24% and specificity 91.58%) of a fused CNN-ResNet network to detect epileptic seizure events from single-lead ECG signals originating from a private database. Finally, Mazumder et al [318] implemented a CNN-LSTM structure for the detection of shockable rhythms in ECG signals from 2 public databases, achieving sensitivity scores between 94.68% and 99.21% and specificity scores between 92.77% and 99.68% for 2- and 8-second time windows, respectively.

## Discussion

### Principal Findings

DL has led to the creation of robust models that could potentially perform fast and reliable clinical diagnoses based on physiological signals. Remarkably, during the past 2 years, at least 230 studies that used DL on ECG data for various clinical applications were identified in the literature, which is a large number for such a short period, regardless of the application domain. This is mainly justified by the fact that DL methods can automatically capture distinctive features from ECG signals based on the trained models that achieve promising diagnostic performance, as shown in Multimedia Appendix 2 [93-322]. This constitutes a significant advantage compared with classical ML methods that perform manual feature selection and feature extraction—2 processes that conventionally require considerable effort and time [323]. Overall, CNN represents the most popular DL architecture and has been identified in most of the reviewed studies (142/230, 60.9% articles). On the contrary, 18.3% (42/230) of studies used LSTM architecture, whereas a ResNet architecture was used in 17.8% (41/230) of cases.

However, training a DL model is not always straightforward. Both architectural design choices and parameter tuning influence model performance; thus, multiple combinations must be considered. Furthermore, the training phase of DL algorithms typically involves complex computations that can be translated into long training times. This requires expensive state-of-the-art computer hardware, including graphics processing units that can dramatically accelerate the total execution time [324].

Another common problem with DL algorithms is overfitting; this occurs when the algorithm fits the noise and therefore performs well on the training set but fails to generalize its predictions to unseen data (ie, the testing set). For this reason, it is necessary to adopt an early stopping strategy during the training phase to prevent further training when the model’s performance on unknown data starts to deteriorate. This is usually done by implementing a separate data set, called the validation set, which most of the time is a small percentage of the training set that is held back from training to provide an unbiased evaluation of the model during training. Moreover, random data splitting can introduce bias; thus, k-fold cross-validation or leave-one-out cross-validation strategies are preferred when training DL models. In addition, it is important that different sets (ie, training, validation, and testing) contain different patients, also known as interpatient data splitting, so that the study’s results are more reliable. As concluded by this review and presented in Multimedia Appendix 2 [93-322], many researchers do not take this into consideration; hence, their results are questionable.

Another critical issue related to overfitting is the distribution of labels or predicted variables in the data set used for model development and validation. For instance, in the BP prediction problem, large stretches of constant BP from the same individual would bias the network toward a constant predictor with minimal error, with the network preferring to memorize patient-identifying features to predict the average BP for a patient rather than those which represent physiological metrics useful in predicting variable BP for the same patient. The resulting errors would be deceptively low if a patient’s nominal BP does not change but, critically, would not be clinically useful in the setting of hypertensive or hypotensive crisis or to guide patient care. None of the assessed papers described the results, indicating that the predicted BP follows meaningful trends.

Recent attention in the medical field to the concept of BP variability [325] rather than clinical spot checks highlights the need for ambulatory BP monitors that are both ergonomic for the patient to increase compliance and comfort, as well as reliable and well validated. A common pitfall in the use of calibrated techniques is that subsequent test data points do not differ significantly from the calibration value and thus yield small errors in prediction, whereas the data are presented as an aggregate pooled correlation plot or Bland-Altman plot with a correlation value that simply reflects the range of BPs across the population rather than patient-specific BP variation [326,327]. In our review of articles using DL for BP prediction, we did not encounter significant attempts to address the issue of BP variability in training data; in fact, many publications explicitly removed data points with hypertensive values or large pulse pressures from their data sets as “artifacts” [93-96,98].

In a calibration-less approach, a narrow range of variation would lead to a low prediction error even when predicting the population mean for each patient. If an ambulatory BP monitoring device plans to use AI-based techniques to measure variability, this variability must be represented in the training set for a model to learn to predict such changes adequately. A way of accomplishing this is to incorporate a variety of BP-modulating activities in the training data, which represent different sources of BP change and corresponding modulations in the feature space. For example, ice pressor tests may increase BP via peripheral vasoconstriction [328], whereas the valsalva maneuver increases chest pressure extrinsically [329] and may modulate input features such as heart rate in opposite ways, reducing the chance that bias-prone DL architectures learn misleading relationships.

In addition to the training and evaluation data, evaluation metrics and cost functions are areas with significant room for improvement. Mean squared error alone can be minimized with a constant predictor if the BP range does not vary significantly. Alternative cost functions such as cosine similarity, which is maximized with constant inputs, contrastive losses, or combinations thereof, have been successful in classification problems in imbalanced, rare event prediction problems such as critical events in patients with COVID-19 [330]. For other promising solutions, it would be prudent to examine similar trend prediction problems in other fields such as stock price movement, where progress has been made using intuitive data preparation and creative representation of the prediction targets, in this case, price changes, to generate trend deterministic predictions [331].

Furthermore, a vast majority of available ECG data sources experience data imbalance. This creates a major problem when trying to predict smaller classes that usually represent rare conditions or diseases that are as important as larger classes when designing health care decision support systems. To solve this problem, several oversampling techniques have been proposed, including random oversampling and undersampling, the synthetic minority oversampling technique [332], the adaptive synthetic sampling technique [333], the generative oversampling method [334], distribution-based balancing [335], and new loss functions such as focal loss [336], which can achieve both prediction error reduction and data imbalance handling. Papers addressing classification frequently use techniques to address class imbalance; however, evidence for such corrections in regression models does not appear as frequently or rigorously.

In addition, DL models are often characterized by black box behavior (lack of interpretability); that is, it is difficult for a human to understand why a particular result is generated by such complex architectures. This is crucial when training models for medical applications, as diagnoses based on unexplained model predictions are not usually accepted by medical experts. A possible solution to this problem is to take advantage of algorithms that are more easily interpretable, such as decision trees [337], additive models [338], attention-based networks [339], and sparse linear models [340], when designing a DL architecture. By contrast, several DL model interpretation approaches have been proposed in this direction, including permutation feature importance [341], partial dependence plots [342], and local interpretable model-agnostic explanations [343]. However, these techniques are rarely used in practice as they require additional time and effort. A useful technique that is used more often when dealing with medical images (and CNNs) is gradient-weighted class activation mapping [344], which makes CNN-based models more transparent by presenting visual explanations for their decisions.

Uncertainty quantification is another common problem associated with DL methods, which has recently drawn the attention of researchers. There are 2 main types of uncertainty: aleatoric (data uncertainty) and epistemic (knowledge uncertainty). It is important to evaluate the reliability and validity of DL methods before they can be tested in real-world applications; thus, uncertainty estimation should be provided. In the past few years, several uncertainty quantification techniques have been proposed, including deep Bayesian active learning [345], Monte Carlo dropout [346], Markov chain Monte Carlo [347], and Bayes by backprop [348].

Moreover, as presented in Multimedia Appendix 1 [54-92], there is no gold standard for data collection. As shown in Multimedia Appendix 2 [93-322], different studies used ECG data with distinct characteristics, namely, the number of leads, signal duration, and sample rate. In addition, many studies used multimodal data, such as photoplethysmograms, arterial BP, polysomnography, and electroencephalograms. Some studies used raw waveforms as input to their models, whereas others precomputed a set of features. This heterogeneity makes it difficult to compare study results; thus, finding the best algorithm is challenging, if not impossible.

Recent advancements [349] in materials and techniques to produce flexible, skin-integrated technology [350] have enabled the development of unique sensors and devices that can simultaneously measure both conventional and novel types of signals from the human body. Small wireless devices [351] such as these can extract continuous ECG; acceleration-based body orientation; physical activity [352]; vibrations such as heart sounds, breath sounds [353]; vocal processes [354]; and photoplethysmogram signals at multiple wavelengths and body locations. This wealth of physiological information that can be measured noninvasively and continuously throughout day-to-day life is potentially a treasure trove of useful insights into health status outside the rigidity of a clinical system. Tools such as DL have emerged as a tantalizing approach to take advantage of such multivariate data in the context of the increased complexity and unpredictability of ambulatory environments. With careful data curation and training approaches, as well as the use of intuitive, well-justified algorithms and network structures, explainable AI can also provide justifications for the use of novel features of underlying physiological relevance. Currently, the use of highly complex and computationally expensive DL models in wearable applications is limited. Generally, raw data are processed in a post hoc fashion after data have been uploaded to cloud servers, limiting real-time feedback. However, recently, there have been developments by chip manufacturers to enable “edge inferencing” by bringing AI-enabling computational acceleration to the low–power-integrated circuit level, opening up the possibilities for low-latency applications of DL algorithms. We strongly believe that the creation of robust DL models that can assist medical experts in clinical decision-making is an important direction for future investigations.

In general, we believe that with this study, we (1) provided a complete and systematic account of the current state-of-the-art DL methods applied to ECG data; (2) identified several ECG data sources used in clinical diagnosis, even some not so widely cited databases; and (3) identified important open research problems and provided suggestions for future research directions in the field of DL and ECG data. Several important relevant review studies have already presented novel DL methods that are used on ECG data [355-357]. Nonetheless, none of them combine all the aforementioned characteristics, which makes this study innovative.

By contrast, the limitations of this study could be summarized as the fact that owing to the enormous number of studies focusing on DL and ECG data, we performed a review based only on articles that have been published in various journals between January 2020 and December 2021.

Although the rationale behind this study was to identify all state-of-the-art DL methods that are applied to ECG data for various clinical applications, in the future, we intend to concentrate our efforts on providing a more complete account of DL methods that make good use of ECG data to address a specific clinical task (ie, congestive heart failure diagnosis).

### Conclusions

In this study, we systematically reviewed 230 recently published articles on DL methods applied to ECG data for various clinical applications. We attempted to group the proposed DL approaches per field of application and summarize the most notable approaches among them. To the best of our knowledge, this is the first study that provides a complete account of the detailed strategy for designing each one of the proposed DL systems by recording the ECG data sources, data preprocessing techniques, model training, evaluation processes, and data splitting strategies that are implemented in each approach. Finally, open research problems and potential gaps were discussed to assess the future of the field and provide guidance to new researchers to design and implement reliable DL algorithms that can provide accurate diagnoses based on ECG data to support medical experts’ efforts for clinical decision-making.

## Appendix

Multimedia Appendix 1 Summary of the major electrocardiogram databases.

Multimedia Appendix 2 Summary of works carried out using deep-learning algorithms and electrocardiogram signals.

## Abbreviations

AF: atrial fibrillation
AI: artificial intelligence
BIH: Beth Israel Hospital
BP: blood pressure
CNN: convolutional neural network
CPSC: China Physiological Signal Challenge
CVD: cardiovascular disease
DBP: diastolic blood pressure
DL: deep learning
DNN: deep neural network
ECG: electrocardiogram
GRU: gated recurrent unit
LSTM: long short-term memory
MIT: Massachusetts Institute of Technology
ML: machine learning
NN: neural network
PTB: Physikalisch Technische Bundesanstalt
ResNet: residual neural network
RNN: recurrent neural network
SBP: systolic blood pressure
