# Supplementary material for: State-of-the-Art Deep Learning Methods on Electrocardiogram Data: Systematic Review
Source: JMIR Med Inform. 2022 Aug 15;10(8):e38454. doi: 10.2196/38454 (PMC9425174; doi:10.2196/38454)
Supplement: Multimedia Appendix 1 [file medinform_v10i8e38454_app1.docx]

# Multimedia Appendix 1: Summary of the major electrocardiogram databases.

| **database** | **enrollment period** | **no. patients** | **no. recordings** | **ECG system** | **duration** | **sample rate** | **content** |
| --- | --- | --- | --- | --- | --- | --- | --- |
| 2011 Physionet/CinC Challenge [54] | unspecified | unspecified | unspecified | 12-lead | 10 s | 500 Hz | ECG quality assessment |
| 2013 Physionet/CinC Challenge [55] | unspecified | unspecified | 447 | 4-lead | 1 min | 1 kHz | fetal QRS and QT interval annotations |
| 2015 PhysioNet/CinC Challenge [56] | unspecified | unspecified | 750 | 2-lead | 5-5.5 min | 250 Hz | 5 alarm types |
| 2017 PhysioNet/CinC Challenge [57] | unspecified | unspecified | 12,186 | single-lead | 9-61 s | 300 Hz | 4 diagnostic classes |
| 2018 PhysioNet/CinC Challenge [58] | unspecified | 1,983 | 1,983 | 1-lead | 7.7 h (mean) | 200 Hz | sleep disorders diagnosis, sleep stages annotation |
| 2020 PhysioNet/CinC Challenge [59] | unspecified | unspecified | 66,361 | 12-lead | 10-1800 s (mean) | 257 Hz-1 kHz | 111 diagnoses or classes |
| Abdominal and Direct Fetal ECG Database [60] | unspecified | 5 | 5 | 5-lead | 5 min | 1 kHz | beat annotations |
| AF Termination Challenge Database [61] | unspecified | 80 | 80 | 2-lead | 1 min | 128 Hz | 3 types of terminating AF |
| Apnea-ECG Database [62] | 1993-1995 and 1998-1999 | 32 | 70 | single-lead | 401-578 min | 100 Hz | sleep apnea |
| BIDMC Congestive Heart Failure Database [63] | unspecified | 15 | 15 | 2-lead | ~20 h | 250 Hz | severe CHF survival |
| CAP Sleep Database [64] | unspecified | 108 | 108 | single-lead | 30 s | 512 Hz | CAP analysis |
| Chapman University and Ningbo First Hospital of Zhejiang University dataset [65] | unspecified | 334 | unspecified | 12-lead | unspecified | 2 kHz | identification of the origins of idiopathic ventricular arrhythmia after catheter ablation |
| Chapman University and Shaoxing People's Hospital Database [66-67] | unspecified | 10,646 | 10,646 | 12-lead | 10 s | 500 Hz | 11 common rhythms and 67 additional cardiovascular conditions |
| Check Your Biosignals Here Initiative (CYBHi) dataset [68] | unspecified | 125 | unspecified | 2-lead | unspecified | 1 kHz | hand palms and fingers ECGs |
| China Physiological Signal Challenge (CPSC) 2018 [69] | unspecified | 6,877 | 6,877 | 12-lead | 6-60 s | 500 Hz | 9 diagnostic classes |
| China Physiological Signal Challenge (CPSC) 2019 [70] | unspecified | 2,000 | 2,000 | 12-lead | 10 s | 500 Hz | beat annotations |
| China Physiological Signal Challenge (CPSC) 2020 [71] | unspecified | 10 | 10 | single-lead | 24 h | 400 Hz | PVC and SBP detection |
| Creighton University Ventricular Tachyarrhythmia Database [72] | unspecified | 35 | 35 | 1-lead | 8 min | 250 Hz | VF onset annotations |
| ECG-ID Database [73] | unspecified | 90 | 310 | 2-lead | 20 s | 500 Hz | annotated beats |
| Fantasia Database [74] | unspecified | 40 | 40 | 3-lead | 120 min | 250 Hz | beat annotations |
| Georgia 12-Lead ECG Challenge Database [59] | unspecified | 15,742 | 20,678 | 12-lead | 10 s | 500 Hz | 6 classes |
| Long Term AF Database [75] | unspecified | 84 | 84 | 2-lead | 24-25 h | 128 Hz | 2 diagnostic classes |
| MIMIC Database [76] | 1994-1996 | 93 | 121 | 1-3 leads | 1-80 h | 500 Hz | multiple diagnoses |
| MIMIC II Waveform Database [77] | 2001-2017 | ~13,500 | 67,830 | 12-lead | unspecified | 125 Hz | multiple diagnoses in ICU |
| MIMIC-III Waveform Database [78-79] | unspecified | 30,000 | 67,830 | 1-lead | unspecified | 125 Hz | ICU monitoring |
| MIT-BIH Arrhythmia Database [80] | 1975-1979 | 47 | 48 | 2-lead | 30 min | 360 Hz | 17 diagnostic classes |
| MIT-BIH Atrial Fibrillation Database [81] | 1983 | 23 | 23 | 2-lead | 10 h | 250 Hz | 4 diagnostic classes |
| MIT-BIH Malignant Ventricular Arrhythmia Database [82] | unspecified | 22 | 22 | 1-lead | 30 min | 250 Hz | 15 rhythm labels |
| MIT-BIH Noise Stress Test Database [83] | unspecified | unspecified | 15 | 2-lead | 30 min | 360 Hz | noisy ECG annotation |
| MIT-BIH Normal Sinus Rhythm Database [54] | unspecified | 18 | 18 | 2-lead | 23-26 h | 128 Hz | no significant arrhythmias |
| MIT-BIH ST Change Database [84] | unspecified | unspecified | 28 | 2-lead | unspecified | 360 Hz | beat labels |
| PAF Prediction Challenge Database [85] | unspecified | 48 | 50 | 2-lead | 5-30 min | 128 Hz | PAF episodes |
| PTB Diagnostic ECG Database [86] | unspecified | 290 | 549 | 12-lead+Frank | unspecified | 1 kHz | 9 diagnostic classes |
| PTB-XL ECG dataset [87] | 1989-1996 | 18,885 | 21,837 | 12-lead | 10 s | 500 Hz | 5 diagnostic superclasses and 24 subclasses |
| QT Database [88] | unspecified | unspecified | 105 | 2-lead | 15 min | 250/360 Hz | annotated beats |
| Smart Health for Assessing the Risk of Events via ECG (SHAREE) Database [89] | 2012-2013 | 139 | 139 | 3-lead | 24 h | 128 Hz | vascular events development risk assessment |
| St Petersburg INCART 12-lead Arrhythmia Database [54] | unspecified | 32 | 75 | 12-lead | 30 min | 257 Hz | 10 diagnoses |
| St. Vincent's University Hospital / University College Dublin Sleep Apnea Database [90] | unspecified | 25 | 25 | 3-lead | 5.9–7.7 h | unspecified | sleep stages, onset time and duration of respiratory events |
| Sudden Cardiac Death Holter Database [82] | mainly in 1980s | 23 | 23 | unspecified | 4-25 min | 250 Hz | 3 diagnostic classes |
| TELE ECG Database [91] | unspecified | 120 | 250 | single-lead | unspecified | 500 Hz | QRS annotations |
| WCS dataset [92] | unspecified | 18 | 18 | 1-lead | 5 min | 128 Hz | 6 different physiological measures collected with wearable devices |
