# Supplementary material for: State-of-the-Art Deep Learning Methods on Electrocardiogram Data: Systematic Review
Source: JMIR Med Inform. 2022 Aug 15;10(8):e38454. doi: 10.2196/38454 (PMC9425174; doi:10.2196/38454)
Supplement: Multimedia Appendix 2 [file medinform_v10i8e38454_app2.docx]

# Multimedia Appendix 2: Summary of works carried out using deep-learning algorithms and electrocardiogram signals.

| **medical application** | **author** | **year published** | **medical task** | **data** | **data preprocessing** | **splitting strategy** | **DL algorithm** | **performance** |
| --- | --- | --- | --- | --- | --- | --- | --- | --- |
| ***BP estimation*** | Li et al. [93] | 2020 | continuous BP estimation | ECG, PPG and ABP recordings from the MIMIC-II database | low-frequency removal using DFT normalization | 10-fold cv | BiLSTM+ResNet | MAE = 6.726 mmHg, RMSE = 8.051 mmHg for SBP MAE = 2.516 mmHg, RMSE = 3.998 mmHg for DBP |
|  | Miao et al. [94] | 2020 | continuous BP measurement | 897,743 heartbeats of ECG, PPG and BP signals from 1,711 patients | noise filtering segmentation normalization | train = 65%, val = 10%, test = 25% | ResNet+LSTM | ME = −0.22 ± 5.82 mmHg for SBP ME = −0.57 ± 4.39 mmHg for MAP ME = −0.75 ± 5.62 mmHg for DBP |
|  | Hill et al. [95] | 2021 | ABP imputation | ECG, PPG and ABP recordings from the MIMIC-III database and 115 ICU patients from the UCLA Health hospital system | downsampling noise filtering normalization segmentation exclusion | train = 90%, val = 10% | 1D V-Net | RMSE = 5.823 mmHg for ABP waveform ME = 2.398 ± 5.623 mmHg for SBP ME = − 2.497 ± 3.785 mmHg for DBP |
|  | Paviglianiti et al. [96] | 2021 | ABP prediction | ECG, PPG and ABP signals from the MIMIC database | NaN replacement deletion denoising standardization normalization | LOOCV | ResNet+LSTM | MAE = 4.118 mmHg for SBP  MAE = 2.228 mmHg for DBP |
|  | Fan et al. [97] | 2021 | continuous BP estimation | 21,422 ECG and ABP signals from the MIMIC II Waveform Database | noise filtering segmentation ground truth labels extraction | train = 80%, val = 10%, test = 10% | BiLSTM | 0.12 ± 10.83 mmHg for SBP 0.13 ± 5.90 mmHg for DBP 0.08 ± 6.47 mmHg for MAP  (performance measured as ME ± RMSE) |
|  | Jeong and Lim [98] | 2021 | continuous BP estimation | ECG, PPG and ABP signals from the MIMIC database | deletion interpolation noise filtering peak detection segmentation zero padding normalization difference | train = 80%, test = 20% | CNN-LSTM | ME = 0.0 ± 1.6 mmHg for SBP ME = 0.2 ± 1.3 mmHg for DBP |
| ***CVD diagnosis*** | Baalman et al. [99] | 2020 | AF detection | 1,469 ECGs in AF or SR from patients with a history AF | smoothing baseline correction | train = 60%, val = 25%, test = 15% | FNN | acc = 96%, F1 = 94% |
|  | Gao et al. [100] | 2020 | AF detection | 8,528 single-lead ECG signal recordings from the 2017 PhysioNet/CinC Challenge | replication expansion cropping normalization | 4-fold cv | TA-CNN | acc = 82.32% |
|  | Cai et al. [101] | 2020 | AF detection | 16,557 samples of 12-lead ECG recordings from 11,994 subjects | noise filtering standardization | train = 64%, val = 16%, test = 20% + 5-fold cv | DDNN | sen = 99.19%, spe = 99.44%, acc = 99.35% for AF vs. normal sen = 97.04%, spe = 98.63%, acc = 98.21% for AF vs. non-AF sen = 98.85%, spe = 98.38%, acc = 97.74% for AF, normal and other classes |
|  | Shi et al. [102] | 2020 | AF detection | ECG signals from the MIT-BIH Atrial Fibrillation Database and the MIT-BIH Arrhythmia Database | none | 10-fold cv | MIDNN | acc = 97.53%, sen = 100.00%, PPV = 95.29% |
|  | Ghosh et al. [103] | 2020 | AF detection | ECG signals from the MIT-BIH Atrial Fibrillation Database and the MIT-BIH Arrhythmia Database | resampling exclusion | 10-fold cv | H-ELM | sen = 98.77%, spe = 100%, acc = 99.40% |
|  | Hsieh et al. [104] | 2020 | AF detection | 8,528 single-lead ECG recordings from the 2017 PhysioNet/CinC Challenge | histogram-based length normalization | 5-fold cv | 1D-CNN | F1 = 78.2% |
|  | Mousavi et al. [105] | 2020 | AF detection | ECG recordings from the MIT-BIH Atrial Fibrillation Database and 2017 PhysioNet/CinC Challenge | segmentation threshold labelling exclusion | 10-fold cv | BiRNN | sen = 99.08%, spe = 98.54%, acc = 98.81% for AFDB (intrapatient) sen = 90.53%, spe = 79.54%, acc = 82.41% for AFDB (interpatient) sen = 86.02%, spe = 98.62%, acc = 96.98% for CinC2017 |
|  | Tran et al. [106] | 2020 | AF detection | 8,528 single-lead ECG signal recordings from the 2017 PhysioNet/CinC Challenge | RR features RR histogram Poincare plot average beat delineation | 5-fold cv | CNN+LSTM+ResNet | F1 = 80% |
|  | Abdelazez et al. [107] | 2020 | AF detection | ECG recordings from the Long-Term AF Database (training) and the MIT-BIH Atrial Fibrillation Database (testing) | noise filtering segmentation SNR estimation wave detection spectrograms using STFT compression | train = 80%, test = 20% | MobileNetV2 | pre = 80%, AUC = 0.87 (uncompressed) pre = 70%, AUC = 0.78 (50% compression) pre = 70%, AUC = 0.79 (75% compression) pre = 57%, AUC = 0.75 (95% compression) |
|  | Buscema et al. [108] | 2020 | AF diagnosis | ECG signals from MITDB and AFDB | RR distance moving window RR prediction fuzzyfication of RR prediction | train = 50%, test+pred = 50% | SVCm | acc = 95% |
|  | Oster et al. [109] | 2020 | AF identification | ECG recordings from the 2017 PhysioNet/CinC Challenge for training and 77,202 ECG signals from 75,778 individual subjects  (UK Biobank study) for evaluation and testing | peak detection SQI estimation | separate train/test | CNN+LSTM | sen = 75.0%, PPV = 97.5%, F1 = 84.8% |
|  | Lai et al. [110] | 2020 | AF screening | ECG data from 37 AF patients and 18 controls | segmentation exclusion delineation f-wave spectrum | train = 30%, test = 70% | CNN | sen = 93.1%, spe = 93.4%, acc = 93.1% |
|  | Kwon et al. [111] | 2020 | anaemia detection | 57,435 ECGs from 31,898 patients (development dataset), 7,974 ECGs from 7,974 patients (internal validation dataset) and 4,665 ECGs from 4,665 patients (external validation dataset) | artifact removal rearrangement | train = 80%, val = 10%, test = 10% | CNN | AUROC = 0.923 for internal validation AUROC = 0.901 for external validation |
|  | Kwon et al. [112] | 2020 | AR detection | 39,371 ECGs for training, 6,453 ECGs for internal validation and 10,865 ECGs for external validation | none | train = 70%, val = 10%, test = 20% | 2D-CNN | AUC = 0.884 for internal validation using 12-lead ECG AUC = 0.861 for external validation using 12-lead ECG AUC = 0.845 for internal validation using single-lead ECG AUC = 0.821 for external validation using single-lead ECG |
|  | Hsu and Cheng [113] | 2020 | arrhythmia classification | ECG signals from the MIT-BIH Arrhythmia Database | noise filtering local minima search drift removal curve fitting signal to image transformation | train = 19.5%, test = 80.5% | 2D-CNN | acc = 97.8% for 5 ECG classes |
|  | Chen et al. [114] | 2020 | arrhythmia classification | 6,877 12-lead recordings from the China Physiological Signal Challenge (CPSC) 2018 | zero padding | train = 70%, test = 30% + 10-fold cv | CNN | F1 = 0.837 |
|  | Cheng et al. [115] | 2020 | arrhythmia classification | ECG recordings from the MIT-BIH Arrhythmia Database | segmentation resampling compression | train = 80%, test = 20% | 1D-CNN+ResNet | exact match rate = 96.03%, 94.99% and 93.19% for compression ratio = 70%, 80% and 90% respectively |
|  | Lennox and Mahmud [116] | 2020 | arrhythmia classification | ECG recordings from the MIT-BIH Arrhythmia Database | peak detection beat extraction normalization zero padding | train = 95%, test = 5% | DNN | acc = 96.04% |
|  | Chen et al. [117] | 2020 | arrhythmia classification | ECG recordings from the MIT-BIH Arrhythmia Database | segmentation RR interval features extraction | train = 50%, test = 50% (interpatient) | CNN | acc = 94.35% |
|  | Wang et al. [118] | 2020 | arrhythmia detection | 20,036 8-lead medical ECG recordings including 34 arrhythmia categories | noise filtering | 5-fold cv | GoogleNet+ResNet+SeResNet+SeInceptionNet | F1 = 92.38% |
|  | Liang et al. [119] | 2020 | arrhythmia detection | ECG signals from the MIT-BIH Arrhythmia Database, the China Physiological Signal Challenge (CPSC) 2018  and the 2017 PhysioNet/CinC Challenge | normalization | 10-fold cv | CNN+BiLSTM | F1 = 82.6% |
|  | Wang et al. [120] | 2020 | arrhythmia detection | ECG recordings from the China Physiological Signal Challenge (CPSC) 2018 and the 2017 PhysioNet/CinC Challenge | augmentation padding normalization | 5-fold cv | DMSFNet | F1 = 82.8% (CPSC 2018) F1 = 84.1% (CinC 2017) |
|  | Zhang et al. [121] | 2020 | arrhythmia detection | ECG signals from the China Physiological Signal Challenge (CPSC) 2018 | downsampling cropping zero padding | 4-fold cv | STA-CRNN | F1 = 83.5% |
|  | Sanjana et al. [122] | 2020 | arrhythmia detection | ECG recordings from the 2017 PhysioNet/CinC Challenge (TD1), Creighton University Ventricular Tachyarrhythmia (TD2-A),  MIT-BIH Malignant Ventricular Ectopy (TD2-B) and MIT-BIH Arrhythmia (TD3) databases | segmentation | 5-fold cv | RNN, LSTM, GRU, CNN and RSCNN | RNN's acc = 96.87% (TD1-class 0), 90.34% (TD1-class 1), 90.88% (TD2-A), 94.71% (TD2-B-lead I), 94.18% (TD2-B-lead II) and 23.73% (TD3) |
|  | Hata et al. [123] | 2020 | AS classification | 700 ECG data from 592 AS patients and 108 non-AS controls | baseline removal peak detection segmentation resampling signal to image conversion | train = 50%, val = 25%, test = 25% | VGG16+Grad-CAM | acc = 88.6%, sen = 86.4%, pre = 90.5%, F1 = 88.4% |
|  | Hu et al. [124] | 2020 | BBB detection | ECG signals from the China Physiological Signal Challenge (CPSC) 2018 and the MIT-BIH Arrhythmia Database | segmentation | separate train/test | CNN+ResNet | acc = 78.58% |
|  | Butun et al. [125] | 2020 | CAD detection | 95,300 two second and 38,120 5-seclong ECG segments from 40 normal and 7 CAD subjects | upsampling | 5-fold cv | 1D-CapsNet | sen = 99.70%, spe = 98.10%, acc = 99.44% on 2-s ECG segments  sen = 98.75%, spe = 97.97%, acc = 98.62% on 5-s ECG segments |
|  | Kwon et al. [126] | 2020 | cardiac arrest prediction | 47,505 ECGs from 25,672 adult patients | noise filtering cropping rearrangement | train = 70%, val = 30% | CNN | AUROC = 0.913 for internal validation AUROC = 0.948 for external validation |
|  | Yildirim et al. [127] | 2020 | cardiac arrhythmia detection | 12-lead ECG recordings from 10,646 patients | denoising | train = 80%, val = 10%, test = 10% | CNN-LSTM | acc = 92.24% for 7 ECG rhythms  acc = 96.13% for 4 ECG rhythms |
|  | Zhang et al. [128] | 2020 | CVD detection | 277,807 ECG signals | cleaning | train = 94%, test = 6% | CNN | acc = 98.27% for detecting 18 beat types |
|  | Thiagarajan et al. [129] | 2020 | CVD detection | ECG signals from the MIT-BIH Arrhythmia Database and the PTB Diagnostic ECG Database | TCP MFCC resampling segmentation normalization peak detection | dataset standard | DDxNet | acc = 98.5%, F1 = 92.7% for arrhythmia classification pre = 99.9%, sen = 99.7%, acc = 99.9% for MI detection |
|  | Lin et al. [130] | 2020 | dyskalemia detection | 66,321 ECG recordings with corresponding serum potassium concentrations from 40,180 patients | random cropping resampling random variable addition | train = 70%, val = 10%, test = 20% | ECG12Net | sen = 96.7%, spe = 93.3%, AUROC = 0.926 for hypokalemia   sen = 83.3%, spe = 97.8%, AUROC = 0.958 for hyperkalemia |
|  | Jeon et al. [131] | 2020 | ECG beats classification | 109,966 ECG beats from the MIT-BIH Arrhythmia Database and 5,465,546 ECG beats from recording made with the S-Patch device | downsampling noise removal segmentation STFT | train = 90%, test = 10% | RNN  fused RNN | acc = 99.72%  acc = 99.80% |
|  | Niu et al. [132] | 2020 | ECG classification | 100,624 ECG beats from the MIT-BIH Arrhythmia Database | denoising segmentation alignment | train = 50%, test = 50% | CNN | acc = 92.3% for classifying ECG beats into 4 categories |
|  | Rincon et al. [133] | 2020 | ECG classification | 8,528 single-lead ECG signal recordings from the 2017 PhysioNet/CinC Challenge | noise filtering signal to image conversion axis removal cropping resize | train = 80%, val = 10%, test = 10% | MobileNet | acc = 90% for AF detection |
|  | van de Leur et al. [134] | 2020 | ECG classification | 336,835 ECG recordings from 142.040 patients | none | separate train/val | ResNet | AUC = 0.93 |
|  | Niu et al. [135] | 2020 | ECG classification | ECG recordings from the MIT-BIH Arrhythmia Database | SBCX representation | 22-fold cv + separate test | MPCNN | acc = 96.4% F1 = 76.6% (SVEB) F1 = 89.7% (VEB) |
|  | Saadatnejad et al. [136] | 2020 | ECG classification | ECG recordings from the MIT-BIH Arrhythmia Database | class splitting segmentation RR interval features extraction wavelet features extraction | separate train/test | LSTM | F1 = 78.8% (SVEB) F1 = 95.5% (VEB) |
|  | Steenkiste et al. [137] | 2020 | ECG classification | ECG recordings from the MIT-BIH Arrhythmia Database and eECG recordings from a private eECG dataset (26,440 beats/4 classes) | noise filtering peak detection | train = 60%, val = 20%, test = 20% | CNN | acc = 97.7% (MIT-BIH) acc = 92.6% and 97.1% without and with transfer learning respectively (eECG dataset) |
|  | Liu et al. [138] | 2020 | ECG classification | 202,103 ECG images from 194,525 different patients plus 5,000 synthetic positive samples for data augmentation | delineation segmentation grayscaling | train = 89.55%, val = 9.95%, test = 0.5% | VQ-VAE | F1 score was improved by 0-6% for most classes using augmented data |
|  | Vijayarangan et al. [139] | 2020 | ECG classification interpretability | ECG rhythms from the MIT-BIH Arrhythmia Database (MITDB), Long-Term Atrial Fibrillation Database (LTAFDB)  and MIT-BIH Long-Term Database (LTDB) | segmentation | train = 4%, test = 96% | CNN+LSTM | R peaks are crucial for this classification task |
|  | Ribeiro et al. [140] | 2020 | ECG diagnosis | 2,322,513 ECG recordings from 1,676,384 patients | none | train = 98%, val = 2% | DNN | F1 > 80%, spe > 99% |
|  | Zhu et al. [141] | 2020 | ECG diagnosis | 180,940 ECGs from 71,520 patients | none | train = 90%, val = 10% + separate test | CNN | AUROC = 0.983, sen = 86.7%, spe = 99.5% |
|  | Lih et al. [142] | 2020 | ECG diagnosis | lead II ECG signals from 92 controls, 7 CAD patients, 148 MI patients and 15 CHF patients (PTB, Fantasia, INCART, BIDMC) | upsampling segmentation | 10-fold cv | CNN-LSTM | sen = 99.30%, spe = 97.89%, acc = 98.51%, PPV = 97.33% |
|  | Mousavi et al. [143] | 2020 | false arrhythmia alarm reduction | ECG, PPG and ABP recordings from the 2015 PhysioNet/CinC Challenge | normalization segmentation | train = 60%, test = 40% | CNN-LSTM | sen = 93.88%, spe = 92.05% |
|  | Shahin et al. [144] | 2020 | heartbeat classification | ECG beats from the MIT-BIH Arrhythmia Database | normalization standardization | train = 80%, test = 20% | DNN | acc = 87% for 5 ECG beat classes |
|  | Romdhane et al. [145] | 2020 | heartbeat classification | ECG recordings from the MIT-BIH Arrhythmia and INCART databases | segmentation normalization | train = 50%, test = 50% | CNN | acc = 98.41%, F1 = 98.38%, pre = 98.37%, sen = 98.41% |
|  | Li et al. [146] | 2020 | heartbeat classification | ECG recordings from the MIT-BIH Arrhythmia Database | noise removal segmentation | train = 64%, val = 16%, test = 20% | ResNet | sen = 94.54%, spe = 98.14%, acc = 99.38% |
|  | Soh et al. [147] | 2020 | hypertension detection | 18 normal ECG signals (MIT-BIH Normal Sinus Rhythm Database) and 139 hypertensive ECG signals (SHAREE Database) | segmentation denoising | 10-fold cv + LOOCV | CNN | sen = 99.99%, spe = 99.97%, acc = 99.99%, PPV = 99.99% (10-fold CV) sen = 99.99%, spe = 99.92%, acc = 99.99%, PPV = 99.99% (LOOCV) |
|  | Porumb et al. [148] | 2020 | hypoglycemia detection | ECG and glucose recordings from 8 individuals | peak detection heartbeat isolation standardization downsampling | train = 45%, val = 10%, test = 45% | CNN+RNN | sen = 84.7%, spe = 84.5% |
|  | Kwon et al. [149] | 2020 | LVH detection | 21,286 patients (4353 with LVH) with paired ECGs and echocardiograms | cropping normalization noise filtering | train = 80%, val = 20% + separate test | ENN | AUC = 0.880 for internal validation AUC = 0.868 for external validation |
|  | Cho et al. [150] | 2020 | MI detection | 412,461 ECGs from 283,878 patients | cropping rearrangement | train = 75%, val = 10%, test = 15% | VAE | AUROC = 0.880 for internal validation AUROC = 0.854 for external validation |
|  | Makimoto et al. [151] | 2020 | MI detection | ECG recordings from the PTB diagnostic ECG Database | signal to image conversion clipping | train = 70%, val = 15%, test = 15% using 10 different splits | CNN | F1 = 83%, acc = 70% |
|  | Fu et al. [152] | 2020 | MI detection/location | ECG recordings from the PTB Diagnostic ECG Database | noise filtering peak detection segmentation deletion | 5-fold cv | CNN+BiGRU | sen = 99.99%, spe = 99.63%, acc = 99.93% (intrapatient MI detection) sen = 97.10%, spe = 93.34%, acc = 96.50% (interpatient MI detection) sen = 99.02%, spe = 99.10%, acc = 99.11% (intrapatient MI location) sen = 63.97%, spe = 63.00%, acc = 62.94% (interpatient MI location) |
|  | Raghunath et al. [153] | 2020 | mortality prediction | 2,338,833 ECGs from 536,661 patients | resampling | train = 60%, test = 40% | DNN | AUC = 0.876 for predicting 1-year mortality |
|  | Kwon et al. [154] | 2020 | MR detection | ECGs from 38,241 patients (2,973 with significant MR) | noise filtering normalization | train = 90%, val = 10% | CNN | AUROC = 0.816, AUPRC = 0.600 for internal validation AUROC = 0.877, AUPRC = 0.328 for external validation |
|  | Missel et al. [155] | 2020 | VT origin localization | pace-mapping data from 47 patients | noise removal baseline correction resampling | train = 80%, val = 20% + separate test | CNN-VAE | localization error = 5.3 ± 2.6 mm using 5.4 ± 2.5 pacing sites |
|  | Çınar and Tuncer [156] | 2020 | ECG classification | 96 arrhythmic (MIT-BIH), 30 NSR and 36 CHF ECG signals | spectrograms using STFT | random | Alexnet-SVM | acc = 96.77% |
|  | Cho et al. [157] | 2020 | HFrEF screening | 47,318 ECGs from 24,317 patients | STFT | train = 80%, val = 20% | CNN | AUROC = 0.961 using 12-lead ECGs AUROC = 0.929 using single-lead ECGs |
|  | Gumpfer et al. [158] | 2020 | myocardial scar detection | ECG and MRI data from 114 patients | cropping scaling augmentation | 6-fold cv | CNN | AUC = 0.89, sen = 70.0%, spe = 84.3%, acc = 78.0% |
|  | Noseworthy et al. [159] | 2020 | effects of race/ethnicity on DLM | 97,829 patients with paired ECGs and echocardiograms | none | train = 45%, val = 55% | CNN | AUC ≥ 0.931 for detecting low LVEF in 5 different racial/ethnic subgroups |
|  | Han et al. [160] | 2021 | acute MI detection | 1,039,550 ECGs from 447,445 patients | downsampling | train = 68%, val = 12%, test = 20% | ResNet | AUROC = 0.880 for 12-lead sets AUROC = 0.858 for 4-lead sets AUROC = 0.845 for 3-lead sets AUROC = 0.813 for 2-lead sets AUROC = 0.768 for single-lead sets |
|  | Ivaturi et al. [161] | 2021 | AF detection | 8,528 single-lead ECG recordings lasting from 9 s to 60 s from the 2017 PhysioNet/CinC Challenge | segmentation periodicity normalization | 5-fold cv | MobileNet | acc = 84.38% for classifying 4 ECG types |
|  | Baek et al. [162] | 2021 | AF detection | 2,412 12‑lead ECGs | signal reweighting | train = 70%, val = 10%, test = 20% | LSTM | AUROC = 0.75, sen = 77%, spe = 72%, F1 = 74%, acc = 71.2% |
|  | Radhakrishnan et al. [163] | 2021 | AF detection | ECG signals from 4 public databases (2017 PhysioNet/CinC Challenge, MIT-BIH Arrhythmia Database, MIT-BIH Atrial Fibrillation Database and 2004 PhysioNet/CinC Challenge) | noise filtering segmentation chirplet transform time-frequency plot | 10-fold cv | 2D-BiLSTM | sen = 99.17%, spe = 99.18%, acc = 99.18% |
|  | Tutuko et al. [164] | 2021 | AF detection | ECG signals from 9 public databases (MIT-BIH Atrial Fibrillation Database, 2017 PhysioNet/CinC Challenge, China Physiological Signal Challenge (CPSC) 2018, Long Term AF Database, PAF Prediction Challenge Database, AF Termination Challenge Database, Fantasia Database, MIT-BIH Arrhythmia Database and Chapman University and Shaoxing People’s Hospital ECG database and two ECG databases from an Indonesian Hospital | noise removal segmentation | train = 60%, val = 10%, test = 30% | 1D-CNN | sen = 98.97%, spe = 98.97%, acc = 98.94% for N vs. AF sen = 93.65%, spe = 96.92%, acc = 96.36% for N, AF and Non-AF |
|  | Salinas-Martínez et al. [165] | 2021 | AF detection | ECG recordings from the Long Term AF and MIT-BIH Normal Sinus Rhythm Databases for training and validation and  from the MIT-BIH Atrial Fibrillation, MIT-BIH Arrhythmia, Monzino-AF Databases for testing | upsampling noise filtering thresholding | 3-fold cv | 2D-CNN | sen > 80.65% for AF episodes shorter than 15s sen > 89.66% for AF episodes shorter than 30s |
|  | Seo et al. [166] | 2021 | AF detection | ECGs from the Long-Term Atrial Fibrillation, MIT-BIH Atrial Fibrillation and the MIT-BIH Arrhythmia databases | downsampling standardization segmentation | train = 80%, val = 20% | ResNet | acc = 53–92% |
|  | Jo et al. [167] | 2021 | AF detection | 128,399 ECGs (Sejong ECG dataset) for training and internal validation, and 21,837 (PTB-XL), 10,605 (Chapman)  and 8,528 (CinC 2017) ECGs for external validation | cropping noise filtering normalization | train = 70%, val = 5%, test = 25% | ResNet | sen = 98.2%, spe = 97.0% for internal validation sen = 99.9%, spe = 99.9% for external validation |
|  | Zhang et al. [168] | 2021 | AF detection | ECG recordings from 29 patients, 2017 PhysioNet/CinC Challenge and MIT-BIH Atrial Fibrillation Database | resampling segmentation noise filtering normalization | 10-fold cv | 1D-CNN+LSTM | sen = 92.09%, spe = 96.66%, acc = 96.23% (CinC 2017) sen = 96.46%, spe = 94.49%, acc = 95.28% (AFDB) |
|  | Zhang et al. [169] | 2021 | AF detection | ECG signals from the MIT-BIH Atrial Fibrillation Database | segmentation | 10-fold cv | 1D-CNN+ResNet | sen = 99.15%, spe = 99.43%, acc = 99.32% (intrapatient) for 2 ECG rhythms sen = 89.29%, spe = 89.71%, acc = 90.96% (interpatient) for 2 ECG rhythms |
|  | Petmezas et al. [170] | 2021 | AF detection | ECG recordings from the MIT-BIH Atrial Fibrillation Database | noise filtering peak detection segmentation | 10-fold cv | CNN-LSTM | sen = 97.87%, spe = 99.29% |
|  | Nishimori et al. [171] | 2021 | AP localization | 12-lead ECG and chest X-ray data from 294 cases (240 with WPW syndrome and 54 normal) | image to signal conversion image compression image cropping | 5-fold cv | CNN | acc = 80% |
|  | Sawano et al. [172] | 2021 | AR detection | 29,859 paired ECG and echocardiography data (412 AR cases) | none | train = 65%, val = 15%, test = 20% | 2D-CNN+DNN | AUC = 0.802 |
|  | Yang et al. [173] | 2021 | arrhythmia classification | 7,000 12-lead ECG recordings lasting from 9 s to 91 s (the 1st China ECG Intelligence Challenge) | none | train = 90%, test = 10% | CCNN | F1 = 86.5% for classifying 12‑lead ECG data into 9 categories |
|  | Kiyasseh et al. [174] | 2021 | arrhythmia classification | 328 30s ECG recordings from 292 patients and ECG recordings from 3 public databases  (Chapman, PhysioNet 2020 and PhysioNet 2017) | none | random interpatient splitting | CNN | AUC = 0.796, 0.834, 0.731 and 0.664 in 4 different continual learning scenarios |
|  | Che et al. [175] | 2021 | arrhythmia classification | ECGs from 6,877 individuals | noise removal segmentation | train = 90%, test = 10% | CNN+Transformer | F1 = 78.6% |
|  | Jo et al. [176] | 2021 | arrhythmia classification | 86,802 ECGs for training-internal validation (Sejong ECG dataset) and  36,961 ECGs for external validation (PTB-XL, Georgia, Chapman, CPSC) | noise filtering normalization | train = 75%, val = 25% + separate test | NBDT | AUC = 0.976 for internal validation AUC = 0.966 for external validation |
|  | Mousavi et al. [177] | 2021 | arrhythmia classification | ECGs from the MIT-BIH Atrial Fibrillation, 2017 PhysioNet/CinC Challenge and the MIT-BIH Arrhythmia databases | peak detection segmentation vocabulary creation wave assignment wave embedding | 10-fold cv | CNN RNN RNN+Attention | F1 = 64.40%, acc = 72.62% (CinC 2017) sen = 98.08%, spe = 97.84% (MITAF) |
|  | Jang et al. [178] | 2021 | arrhythmia classification | 2,648,100 ECG data points from 26,481 subjects and 10,646 ECG data points from 10,646 subjects | resampling size adjustment noise removal normalization | train = 80%, test = 20% | CAE | F1 = 84.3% (100% bootstrapping) F1 = 83.1% (50% bootstrapping) F1 = 54.3% (25% bootstrapping) |
|  | Jiang et al. [179] | 2021 | arrhythmia classification | 8,528 single-lead ECG data recordings from the 2017 PhysioNet/CinC Challenge | segmentation | 10-fold cv | ResNet+BiLSTM | pre = 86.6%, sen = 85.9%, acc = 86.7%, F1 = 88.0% |
|  | Lu et al. [180] | 2021 | arrhythmia classification | ECG recordings from the MIT-BIH Arrhythmia Database | class merging segmentation  standardization | train = 70%, val = 10%, test = 20% | 1D-CNN | acc = 99.31%, sen = 99.45%, pre = 98.78% |
|  | Lee and Shin [181] | 2021 | arrhythmia classification | ECG recordings from the 2017 PhysioNet/CinC Challenge | peak detection segmentation ECM generation size adaptation | 5-fold cv | BIT-CNN | F1_NAO = 81.75%, F1_NAOP = 76.87% |
|  | Zhang et al. [182] | 2021 | arrhythmia classification | ECG recordings from the China Physiological Signal Challenge (CPSC) 2018 | downsampling cropping zero padding | 10-fold cv | CNN+BiGRU+Attention | F1 = 85.5% |
|  | Luo et al. [183] | 2021 | arrhythmia classification | ECG recordings from the MIT-BIH Arrhythmia Database | segmentation standardization | 10-fold cv | CNN+LSTM+GRU | sen = 99.58%, acc = 99.01% |
|  | Zhang et al. [184] | 2021 | arrhythmia classification | ECG recordings from the China Physiological Signal Challenge (CPSC) 2018 for training/testing,  and the PTB-XL ECG dataset and Georgia 12-Lead ECG Challenge Database for testing | downsampling segmentation mapping into RP-based images normalization | 5-fold cv | ResNet | F1 = 85.21% (CPSC) F1 = 88.62% (PTB-XL) F1 = 85.29% (Georgia) |
|  | Wang et al. [185] | 2021 | arrhythmia classification | ECG recordings from the MIT-BIH Arrhythmia Database | segmentation normalization | 5-fold cv + train = 50%, test = 50% (interpatient) | AE+1D-CNN | acc = 99.94% for 6 ECG rhythms |
|  | Chang et al. [186] | 2021 | arrhythmia classification | 65,932 12-lead ECG waveform data signals from 38,899 patients | noise filtering | separate train/test | BiLSTM | acc > 98.2% for all 12 heart rhythms |
|  | Elul et al. [187] | 2021 | arrhythmia detection | ECG recordings from the Normal Sinus Rhythm, Long-Term Atrial Fibrillation, MIT-BIH Arrhythmia databases (training)  and the MIT-BIH Atrial Fibrillation, Telemetric and Holter ECG Warehouse databases (testing)  and additional ECG data from seven datasets (extended testing) | removal detrend clipping scaling segmentation resampling PSD estimation STA | train = 50%, test = 50% + separate testing | TCNN | acc = 96% for normal rhythm |
|  | Nannavecchia et al. [188] | 2021 | arrhythmia detection | ECG recordings from the MIT-BIH Arrhythmia Database | downsampling segmentation | train = 40%, val = 15%, test = 45% + train = 65%, test = 35% | 1D-CNN | sen = 87.79%, acc = 89.51%, F1 = 86.78% for detecting 21 anomalies |
|  | Yoo et al. [189] | 2021 | arrhythmia detection | ECG recordings from the China Physiological Signal Challenge (CPSC) 2018 | normalization zero padding | train = 76%, val = 4%, test = 20% + 5-fold cv | CNN | F1 = 81.2% |
|  | Mori et al. [190] | 2021 | ASD diagnosis | 1,192 ECG recordings from 364 ASD patients and 828 controls | cropping grayscale conversion | 5-fold cv | CNN-LSTM | sen = 76%, spe = 96%, acc = 89%, PPV = 88%, F1 = 81% |
|  | Liu et al. [191] | 2021 | Brugada syndrome diagnosis | 276 type 1 Brugada ECGs and 276 randomly retrieved non-Brugada type ECGs | noise filtering | 5-fold cv | CNN+BiLSTM | sen = 88.4%, spe = 89.1%, AUC = 0.96 |
|  | Jahmunah et al. [192] | 2021 | CAD, MI and CHF detection | lead II ECG signals from 92 healthy controls, 7 CAD, 148 MI and 15 CHF patients (PTB Diagnostic ECG Database, Fantasia Database,  St Petersburg INCART 12-lead Arrhythmia Database, BIDMC Congestive Heart Failure Database) | upsampling segmentation | 10-fold cv | CNN  GaborCNN | sen = 99.27%, spe = 99.67%, acc = 99.55%  sen = 98.74%,spe = 99.46%, acc = 98.74% |
|  | Bender et al. [193] | 2021 | CVD detection | ECG recordings from 10 LBBB patients and 19 AF patients | resampling zero padding rescaling | unspecified | DNN | F1 = 100% for LBBB detection F1 = 91.9% for AF detection |
|  | Fu et al. [194] | 2021 | CVD detection | 15,437 anonymous ECG recordings collected from several tertiary hospitals in China used for model testing | noise filtering | unspecified | CNN+RNN | acc = 81.57%, F1 = 82.33%, AUC = 0.9011 for reverse lead detection AUC > 0.98 for 17 out of 20 ECG rhythms |
|  | Dai et al. [195] | 2021 | CVD detection | ECG signals from the PTB Diagnostic ECG Database | normalization | 10-fold cv | 1D-CNN | sen = 99.04%, spe = 99.87%, acc = 99.59% for 1s ECGs sen = 99.48%, spe = 99.93%, acc = 99.80% for 2s ECGs sen = 99.52%, spe = 99.95%, acc = 99.84% for 3s ECGs |
|  | Deevi et al. [196] | 2021 | ECG beat classification | ECG recordings from the MIT-BIH Arrhythmia Database (training/testing) and the CPSC 2018 dataset (testing) | zero padding downsampling noise addition segmentation | train = 80%, val = 10%, test = 10% | 1D U-Net | pre = 98.42%, sen = 98.42%, acc = 98.42% (without denoising) pre = 99.53%, sen = 99.53%, acc = 99.53% (with denoising) pre = 89.51%, sen = 89.51%, acc = 89.51% (CPSC 2018) |
|  | Chen et al. [197] | 2021 | ECG classification | 19,253 ECGs recorded at the Kaohsiung Medical University Hospital (KMUH) 6,877 12-lead ECG recordings (CPSC 2018 dataset) | grayscaling deletion thinning cutting digitization | 5-fold cv + separate val | CNN+ResNet+LSTM | acc = 96.02% (KMUH) acc = 94.07% (CPSC 2018) |
|  | Wang et al. [198] | 2021 | ECG classification | ECG signals from the MIT-BIH Arrhythmia Database and the PTB-XL ECG dataset | resampling segmentation normalization peak detection zero padding | train = 80%, val = 20% | CNN | acc = 98.64%, sen = 96.60%, spe = 99.15%, F1 = 96.64% (MIT-BIH) AUC = 0.9314, Fmax = 0.8507 (PTB-XL) |
|  | Wang et al. [199] | 2021 | ECG classification | ECG signals from the MIT-BIH Arrhythmia Database | noise filtering segmentation interval extraction | train = 50%, test = 50% | CNN | PPV = 70.75%, sen = 67.47%, F1 = 68.76%, acc = 98.74% |
|  | Pokaprakarn et al. [200] | 2021 | ECG classification | ECG recordings from a study in University of Virginia (UVA) Heart Station and the MIT-BIH Arrhythmia Database | normalization peak detection spectrograms using STFT exclusion | train = 64%, val = 16%, test = 20% + separate test | CNN-LSTM | F1 = 89% |
|  | Weimann and Conrad [201] | 2021 | ECG classification | ECG recordings from the 2017 PhysioNet/CinC Challenge, PTB-XL ECG dataset and the China Physiological Signal Challenge (CPSC) 2018 | standardization downsampling zero padding | train = 75%, val = 5%, test = 20% | ResNet | F1 = 77.9% (CinC 2017) AUC = 0.962 (PTB-XL) AUC = 0.961 (CPSC) |
|  | Zhang et al. [202] | 2021 | ECG diagnosis | ECG recordings from the China Physiological Signal Challenge (CPSC) 2018 | cropping zero padding | 10-fold cv | 1D-CNN+ResNet | sen = 81.2%, acc = 96.6%, F1 = 81.3% |
|  | Mishra et al. [203] | 2021 | ECG digitization/classification | 3,200 ECG samples (scanned + camera captured + machine-generated) | signal to image conversion cropping binarization | separate train/test | DNN+CNN | acc = 97% (threshold calculation) acc = 94.4% (arrhythmia classification) |
|  | van de Leur et al. [204] | 2021 | ECG features identification | 12-lead ECG recordings from 86 PLN mutation carriers and 1,720 controls | median beats extraction | train = 80%, test = 20% + 8-fold cv | DNN | AUC = 0.95, sen = 82%, spe = 93% |
|  | Zhang et al. [205] | 2021 | ECG heartbeat classification | ECG recordings from the MIT-BIH Arrhythmia Database | segmentation resampling substraction of average | train = 80%, val = 20% | adversarial CNN | acc = 94.7% sen = 78.8%, pre = 90.8% (SVEB) sen = 92.5%, pre = 94.3% (VEB) |
|  | Ammour et al. [206] | 2021 | ECG heartbeat classification | ECG recordings from the MIT-BIH Arrhythmia, INCART and SVDB databases | segmentation resampling normalization | train = 80%, test = 20% | CNN+DenseNet169 | acc = 98.40% (MIT-BIH) acc = 87.70% (INCART) acc = 88.46% (SVDB) |
|  | Wu et al. [207] | 2021 | ECG signal classification | ECG beats from the MIT-BIH Arrhythmia Database | wavelet denoising segmentation enhancement | 10-fold cv | CNN | sen = 97.05%, spe = 99.35%, acc = 97.41% |
|  | Ma et al. [208] | 2021 | ECG signal classification | ECG signals from the MIT-BIH Atrial Fibrillation Database | denoising standardization segmentation | 5-fold cv | iDCCN | sen = 98.79%, spe = 99.04%, acc = 98.65% |
|  | Siontis et al. [209] | 2021 | HCM detection | ECG and echocardiogram data from 300 children and adolescents with HCM and 18,439 non-HCM controls | exclusion | separate train/val | CNN | AUC = 0.98, sen = 92%, spe = 95% |
|  | Huang et al. [210] | 2021 | heartbeat classification | ECG beats from the MIT-BIH Arrhythmia Database | denoising QRS complex detection heartbeat segmentation | train = 60%, test = 40% (interpatient) | CNN-LSTM-RF | acc = 98.93%, pre = 96.92%, sen = 96.46%, spe = 99.33% for classifying ECG beats into 5 categories |
|  | Zhang et al. [211] | 2021 | heartbeat classification | ECG recordings from the MIT-BIH Arrhythmia Database | noise filtering R wave detection segmentation signal to diagram using HT-WVD | train = 70%, val = 20%, test = 10% | ResNet-101 | sen = 91.36%, spe = 99.85%, acc = 99.75% |
|  | Wang et al. [212] | 2021 | hypokalemia screening | 6,904 ECGs and 1,726 ECGs as development and internal validation, and 1,278 ECGs as external validation data | none | train = 80%, val = 20% | CNN | AUC = 0.80, sen = 71.43%, spe = 77.15% for internal validation AUC = 0.77, sen = 70.01%, spe = 69.14% for external validation |
|  | Paragliola and Coronato [213] | 2021 | identification of hypertension | 24 h ECG recordings of 139 hypertensive subjects | fragmentation | 10-fold cv | LSTM+CNN+DNN | sen = 97%, spe = 97%, acc = 98% |
|  | Sun et al. [214] | 2021 | LVD screening | 26,786 ECG‐TTE pairs | pairing exclusion | train = 80%, val = 10%, test = 10% | CNN | sen = 69.2%, spe = 70.5%, acc = 73.9% |
|  | Attia et al. [215] | 2021 | LVSD detection | ECG signals from 4,277 subjects (26 with LVSD) | noise filtering exclusion truncation | none | CNN | AUC = 0.82, sen = 26.9%, spe = 97.4%, acc = 97.0% |
|  | Bigler and Seiler [216] | 2021 | myocardial ischemia detection | 893 icECGs from 228 patients | signal averaging signal to image conversion augmentation | train = 80%, val = 20% | CNN | AUC = 0.924, sen = 93%, spe = 92% |
|  | Raghunath et al. [217] | 2021 | new-onset AF prediction | 1.6 million resting 12-lead ECG recordings from 431,000 patients | resampling | train = 65%, test = 35% | DNN | AUROC = 0.85, AUPRC = 0.22 |
|  | Yang et al. [218] | 2021 | premature beats detection | ECG recordings from the MIT-BIH Arrhythmia and the China Physiological Signal Challenge (CPSC) 2020 databases | resampling segmentation | 5-fold cv | 1D-CNN+ResNet | F1 = 92.6% for PVC detection F1 = 72.2% for SPB detection |
|  | Yu et al. [219] | 2021 | PVC detection | ECG signals from the MIT-BIH Arrhythmia Database | segmentation | train = 50%, test = 50% | 1D-CNN | sen = 97.45%, spe = 99.87%, acc = 99.7% |
|  | Naz et al. [220] | 2021 | VA detection | ECG data from the MIT-BIH Malignant Ventricular Ectopy, Creighton University Ventricular Tachyarrhythmia and MIT-BIH Arrhythmia databases | signal to image conversion segmentation reshaping | 10-fold cv | AlexNet+VGG19+InceptionV3 | acc = 97.6% |
|  | Petryshak et al. [221] | 2021 | PVC identification | ECG recordings from the MIT-BIH Arrhythmia Database, MIT-BIH Supraventricular Arrhythmia Database and China Physiological Signal Challenge (CPSC) 2020 | resampling noise filtering normalization augmentation | 4-fold cv | U-Net+InceptionTime | acc = 96.5% (MIT-BIH) acc = 96.05% (SVDB) |
|  | Sabut et al. [222] | 2021 | VTA detection | ECG recordings from the Creighton University Ventricular Tachyarrhythmia Database and the MIT-BIH Malignant Ventricular Arrhythmia Database | noise removal standardization | unspecified | DNN | sen = 98.8%, spe = 99.3%, acc = 99.2% |
|  | Liu et al. [223] | 2021 | AD diagnosis | 10-s ECG recordings and CXR images from 43,473 patients | cropping | 5-fold cv | ECG12Net | AUC = 0.882, 0.960 and 0.813 for detecting AD, AD type A and AD type B respectively |
|  | Li et al. [224] | 2021 | ECG classification | ECG signals from the MIT-BIH Arrhythmia Database | segmentation downsampling normalization pairing | unspecified | CNN | acc = 92.42% |
|  | Liu et al. [225] | 2021 | ECG classification | ECG signals from the MIT-BIH Arrhythmia Database | oversampling noise addition | train = 100% | CNN | acc = 80.52% and 60.99% for noise level of 40% and 50% respectively |
|  | Liu et al. [226] | 2021 | acute MI detection | 12-lead ECG recordings from 25,002 (training) and 14,296 (validation) patients | unspecified | separate train/val | ECG12Net | sen = 93.6% for STEMI sen = 66.4% for non-STEMI |
|  | Krasteva et al. [227] | 2021 | AF detection | ECG recordings from the 2017 PhysioNet/CinC Challenge | feature extraction | 5-fold cv | DenseNet-3 | F1 = 79.97% |
|  | Ramesh et al. [228] | 2021 | AF classification | ECG recordings from the MIT-BIH Arrhythmia, MIT-BIH Atrial Fibrillation and MIT-BIH Normal Sinus Rhythm Databases and unseen PPG signals | segmentation filtering downsampling delineation HRV extraction standardization | 5-fold cv + separate test | CNN | acc = 95.50%, sen = 94.50%, spe = 96.00% (5-fold cv) acc = 95.10%, sen = 94.60%, spe = 95.20% (unseen PPG) |
|  | Xie et al. [229] | 2021 | ECG classification | 12-lead ECG recordings from the China Physiological Signal Challenge (CPSC) 2018 | removal downsampling padding | train = 60%, val = 20%, test = 20% | CNN-DCN | acc = 86.3% |
|  | Liu et al. [230] | 2021 | ECG classification | ECG recordings from the China Physiological Signal Challenge (CPSC) 2018, the PTB Diagnostic ECG Database, the PTB-XL ECG dataset, the Georgia 12-Lead ECG Challenge Database, the Chapman University and Shaoxing People’s Hospital ECG database and the Ningbo dataset | resampling noise removal normalization truncation padding | 5-fold cv | 1D ResNet | CWAcc score = 0.641 ± 0.009 |
|  | Ullah et al. [231] | 2021 | arrhythmia detection | ECG recordings from the MIT-BIH Arrhythmia Database and the PTB Diagnostic ECG Database | segmentation scaling standardization | train = 80%, test = 20% | CNN, CNN+LSTM, CNN+LSTM+Attention | acc = 99.12% (CNN) acc = 99.3% (CNN+LSTM) acc = 99.29% (CNN+LSTM+Attention) |
|  | Tadesse et al. [232] | 2021 | MI detection | 10s 12-lead ECG signals from 17,381 patients (11,853 MI and 5,528 Normal cases) | exclusion noise filtering segmentation spectrograms using FT fusion | 5-fold cv | GoogLeNet, MnasNet, LSTM, Dense | AUROC = 96.7%, and 82.9%, 68.6% and 73.8% for Normal cases, and Acute, Recent and Old onset cases of MI respectively |
|  | Adedinsewo et al. [233] | 2021 | cardiomyopathy detection  during pregnancy | 12-lead ECG recordings from 1,807 pregnant or postpartum women | unspecified | test = 100% | CNN | AUC = 0.92 for LVEF ≤ 35% AUC = 0.89 for LVEF < 45% AUC = 0.87 for LVEF < 50% |
|  | Chen et al. [234] | 2021 | HF diagnosis | single-lead ECG recordings from the MIMIC-III database | segmentation | unspecified | CNN | sen = 97.7%, spe = 97.4%, acc = 97.5% |
|  | Akbilgic et al. [235] | 2021 | HF prediction | 10s 12-lead ECG recordings from 14,613 subjects (13,810 controls and 803 HF patients) | none | train = 80%, test = 20% + 5-fold cv | CNN-ResNet | AUC = 0.756 |
|  | Khurshid et al. [236] | 2021 | AF prediction | 12-lead ECG signals from 45,770 individuals for training and 83,162 individuals for testing | resampling zero padding | train = 80%, val = 20% + separate test | CNN | AUROC = 0.705-0.823 |
|  | Gibson et al. [237] | 2021 | STEMI detection | 8,511 ECG recordings (4,255 STEMI and 4,256 non-STEMI) | QRS detection segmentation | train = 90%, test = 10% | CNN | sen = 96.3%, spe = 96.8%, acc = 96.5% for STEMI detection using 12-lead ECGs sen = 82.8%-88.1%, spe = 83%-94.5%, acc = 83.5%-90.5% for STEMI detection using single-lead ECGs |
|  | Zhang et al. [238] | 2021 | AF detection | ECG recordings from the China Physiological Signal Challenge (CPSC) 2018 (training/validation) and the 2017 PhysioNet/CinC Challenge (testing) | segmentation filtering standardization | 10-fold cv + separate test | CNN | acc = 99.84%, pre = 99.89%, sen = 99.65%, spe = 99.98%, F1 = 99.54% |
|  | Bizzego et al. [239] | 2021 | heartbeat detection | ECG recordings from the MIT-BIH Normal Sinus Rhythm Database, the MIT-BIH Long-Term ECG Database, the MIT-BIH Arrhythmia Database and the WCS dataset | segmentation resampling | train = 66%, test = 33% | CNN | MCC = 0.690-0.935 |
|  | Li et al. [240] | 2021 | CAD detection | ECG and PPG signals from 60 non-CAD and 135 CAD subjects | noise filtering segmentation standardization spectra using FT S transform images MFCC images | 5-fold cv | CNN | acc = 96.51%, sen = 99.37%, spe = 90.08% |
|  | Li et al. [241] | 2021 | arrhythmia classification | ECG signals from the MIT-BIH Arrhythmia Database | segmentation re-labelling denoising standardization | train = 25%, val = 25%, test = 50% | ResNet | sen = 94.54%, spe = 80.80% for normal segments sen = 35.22%, spe = 98.83% for SVEB segments sen = 88.35%, spe = 94.92% for VEB segments |
|  | Lai et al. [242] | 2021 | optimal lead selection | 6,877 12-lead ECG recordings from the China Physiological Signal Challenge (CPSC) 2018 and 3,998 12-lead ECG recordings from the 2020 PhysioNet/CinC Challenge | labels merge | train = 80%, val = 10%, test = 10% | ResNet-LSTM | F1 = 76.4% for CPSC 2018 and F1 = 54.7% for CinC 2020 using leads II, aVR, V1, V4 |
|  | Tzou et al. [243] | 2021 | PAF prediction | single-lead ECG and neuECG recordings from 8 adult PAF patients | amplification filtering exclusion | 10-fold cv | CNN | acc = 81%, F1 = 81%, sen = 81%, spe = 80% using ECG acc = 89%, F1 = 88%, sen = 88%, spe = 89% using neuECG |
|  | Bollepalli et al. [244] | 2021 | arrhythmia detection | 4-channel ECG, and BP and PPG recordings from 410 critical care subjects with diverse medical conditions and ECG recordings from the 2015 PhysioNet/CinC Challenge | filtering R-wave detection exclusion segmentation | 5-fold cv + separate test | CNN | acc = 87.5% (ICU data) acc = 93.9% (CinC 2015) |
|  | Malik et al. [245] | 2021 | ECG classification | ECG recordings from the MIT-BIH Arrhythmia Database | downsampling partitioning | train = 40%, test = 60% | 1D Self-ONN | acc = 98%, F1 = 76.6% for SVEB detection acc = 99.04%, F1 = 93.7% for VEB detection |
|  | Luo et al. [246] | 2021 | ECG arrhythmia classification | ECG recordings from the 2017 PhysioNet/CinC Challenge and the China Physiological Signal Challenge (CPSC) 2018 | denoising using DWT label removal segmentation resampling concatenation zero padding | separate train/test | DNN | F1 = 85.2%-87.5% |
|  | Lee et al. [247] | 2021 | arrhythmia detection | ECG recordings from the China Physiological Signal Challenge (CPSC) 2018, the PTB-XL ECG dataset and the Chapman University and Shaoxing People’s Hospital ECG database | noise filtering masking smoothing | train = 70%, val = 15%, test = 15% | ResNet | best F1 = 81.22%, 72.58% and 89.86 for CPSC 2018, PTB-XL and Shaoxing respectively |
|  | Rasmussen et al. [248] | 2021 | AF detection | ECG recordings from the MIT-BIH Atrial Fibrillation Database | segmentation re-labelling balancing via downsampling filtering downsampling | train = 90%, test = 10% | ResNet | acc = 98.7% using 5% labeled data |
|  | Park et al. [249] | 2021 | ECG classification | 13,241 10s 12-lead ECG recordings | resampling via linear interpolation min-max normalization index mapping | train = 64%, val = 16%, test = 20% | SE-ResNet | F1 = 98.7%, 98.2%, 95.1% and 97.4% for normal, AF, PAC and PVC respectively |
|  | Vaid et al. [250] | 2021 | RVD and LVD identification | 715,890 paired ECGs from 147,636 patients for prediction of LVEF and 761,510 paired ECGs from 148,227 patients for prediction of RV status | noise filtering exclusion | separate train/test | DenseNet-201, Efficientnet B4, ResNet-50 | AUC = 0.94 for LVEF ≤ 40% AUC = 0.73 for 40% < LVEF ≤ 50% AUC = 0.87 for LVEF > 50% AUC = 0.84 for RV outcome prediction |
| ***ECG analysis*** | Teplitzky et al. [251] | 2020 | ECG annotation | 20,932 ECG recordings from 11,008 patients (training) 515 ECGs from 505 patients along with ECGs from the MIT-BIH and AFDB databases (validation) | noise filtering resampling | separate train/val | BeatNet+RhythmNet | sen = 99.84%, PPV = 99.78% |
|  | Li et al. [252] | 2020 | ECG digitization | 96 ECG signals with both paper scans and digital ground truth | noise addition | 5-fold cv | U-Net | Dice coefficient = 0.85 |
|  | Cao et al. [253] | 2020 | ECG generation | ECG data from the MIT-BIH Arrhythmia Database, MIT-BIH Atrial Fibrillation Database, MIT-BIH Normal Sinus Rhythm Database,  MIT-BIH Noise Stress Test Database and 2017 Physionet/CinC Challenge | segmentation resampling noise filtering | train = 80%, val = 20% | GFMN | F1 = 82% |
|  | Herraiz et al. [254] | 2020 | ECG segments quality assessment | 96,654 ECG recordings from 3 databases (2017 PhysioNet/CinC Challenge and two proprietary databases) including patients with intermittent AF | scalograms using CWT | train = 80%, test = 20% | 2D-CNN | sen = 86.91%, spe = 91.00%, acc = 88.95%  sen = 95.49%, spe = 85.00%, acc = 91.42%  sen = 97.17%, spe = 92.42%, acc = 94.79% |
|  | Fotiadou et al. [255] | 2020 | fECG denoising | simulated ECG data, ECG data from the Abdominal and Direct Fetal ECG Database and ECGs from the PTB Diagnostic ECG, INCART and QT databases | noise filtering resampling normalization ICA segmentation | separate train/val | CNN | RMSE = 9.9ms for PR interval RMSE = 14ms for QT interval |
|  | Fotiadou and Vullings [256] | 2020 | fECG denoising | 462 six-channel fECG recordings from 462 pregnant women and fECG recordings from the Abdominal and Direct Fetal ECG Database | fECG extraction resampling segmentation normalization | separate train/test | CNN | SNRout = SNRin + 9.5 for SNRin = [-20, 20] dB |
|  | Vo et al. [257] | 2020 | fECG extraction | abdominal ECG recordings from the set-A of the 2013 PhysioNet/CinC Challenge | noise addition motion artifact addition normalization | train = 80%, val = 10%, test = 10% | OctConv | F1 = 91.1% |
|  | Murat et al. [258] | 2020 | heartbeat detection | 100,022 ECG beats from the MIT-BIH Arrhythmia Database | none | train = 80%, val = 10%, test = 10% | CNN-LSTM | acc = 99.26% for 5 ECG classes |
|  | Silva et al. [259] | 2020 | heartbeat detection | ECG recordings from the CYBHi dataset and the MIT-BIH Arrhythmia Database | resampling augmentation | train = 35%, val = 15%, test = 50% | CNN | sen = 95.71%, PPV = 96.77, F1 = 96.00% (CYBHi) sen = 92.98%, PPV = 100.00%, F1 = 96.00% (MIT-BIH) |
|  | Hao et al. [260] | 2020 | pacemaker ECG analysis | 4 paced and 44 non-paced ECGs (MIT-BIH Arrhythmia Database) and 12 paced and 78 non-paced ECGs (Biofourmis ECG database) | CS FFT STFT | separate train/test | CNN | F1 = 97.2% for paced rhythm |
|  | Vijayarangan et al. [261] | 2020 | R peak detection | ECG recordings from the China Physiological Signal Challenge (CPSC) 2019 for training  and from the MIT-BIH Arrhythmia, MIT-BIH ST Change and MIT-BIH Noise Stress Test Databases for testing | segmentation resampling | 3-fold cv | 1D-CNN+ResNet | pre = 99.44%, sen = 99.75%, F1 = 99.65% (MIT-BIH) pre = 99.72%, sen = 99.83%, F1 = 99.78% (ST Change) pre = 98.20%, sen = 94.51%, F1 = 96.32% (NSTDB) |
|  | Zaman and Morshed [262] | 2020 | signal quality estimation | ECG data from the 2011 Physionet/CinC Challenge and PPG data (PPG-BP database) | segmentation re-labelling | unspecified | ANN | pre = 86%, F1 = 92% |
|  | Hicks et al. [263] | 2021 | sex identification | 10-s 12-lead ECGs from 15,606 individuals | none | 5-fold cv | CNN | acc = 88.80% for sex identification |
|  | Gyawali et al. [264] | 2021 | disentangled representation learning | simulated ECG data (SimECG and SimECG-torso datasets) and clinical ECG data collected from routine pace-mapping procedures  on 39 patients who underwent ablation of scar-related VT | none | train = 60%, val = 20%, test = 20% | CNN, VAE, IBP-VAE | disentanglement score = 92.1% |
|  | Jimenez‑Perez et al. [265] | 2021 | ECG delineation | 105 2‑lead ECG recordings (QT Database) | re-annotation exclusion binary mask transformation | 5-fold cv | U‑Net | pre = 90.12%, sen = 98.73% for P wave pre = 99.14%, sen = 99.94% for QRS complex pre = 98.25%, sen = 99.88% for T wave |
|  | Kuznetsov et al. [266] | 2021 | ECG generation | 2,033 10-s ECG signals | segmentation | separate train/test | VAE | MMD = 0.00383 |
|  | Liu et al. [267] | 2021 | ECG quality assessment | ECG data from the 2011 Physionet/CinC Challenge | noise filtering spectrograms using S-transform | 10-fold cv | CNN | acc = 93.09%, F1 = 84.72%, sen = 97.67% |
|  | Seeuws et al. [268] | 2021 | ECG quality assessment | ECG signals from the 2017 PhysioNet/CinC Challenge and two other private databases | noise filtering exclusion resampling | train = 80%, val = 20% | AE-logMSE AE-LLH | sen = 96%, 96% and 94%, spe = 60%, 60% and 60% for normal, AF and other rhythms respectively sen = 93%, 93% and 88%, spe = 70%, 70% and 70% for normal, AF and other rhythms respectively |
|  | Bacoyannis et al. [269] | 2021 | electrocardiographic imaging | 5,200 activation maps and their corresponding BSP | normalization | train = 80%, val = 20% | CVAE | MAE = 11.33 ± 4.10 ms |
|  | Rjoob et al. [270] | 2021 | electrode misplacement detection | ECG recordings for 453 individuals (151 normal, 151 LVH and 151 MI) | multiplication using transformation matrix | none | CNN BiLSTM | acc = 92.3% and 73.5% for misplacement in the second and third intercostal space respectively acc = 93.0% and 74.7% for misplacement in the second and third intercostal space respectively |
|  | Fotiadou et al. [271] | 2021 | fetal HR estimation | fECG data extracted from 28 abdominal recordings from 28 women with a gestational age between 36 and 42 weeks  and 75 1-min noninvasive abdominal signals from the set-A of the 2013 PhysioNet/CinC Challenge | resampling noise filtering signal estimation signal enhancement | train = 60%, val = 20%, test = 20% | CNN-LSTM | PPA = 97.30%  PPA = 99.60% |
|  | Giudicessi et al. [272] | 2021 | QTc assessment | 1,612,617 ECGs from 538,200 patients | noise filtering downsampling average beat extraction | train = 67%, val = 33% | DNN | AUC = 0.97, sen = 80.0%, spe = 94.4% |
|  | Ganapathy et al. [273] | 2021 | R-wave detection | ECG data from 4 public databases (MIT-BIH, INCART, TELE and SDDB) | difference average difference | 5-fold cv | CNN | F1 = 99.75% for MIT-BIH F1 = 95.25% for TELE |
|  | Strodthoff et al. [274] | 2021 | sex identification | 21,837 clinical 12-lead ECG recordings of 10 s length from 18,885 patients (PTB-XL) and 6,877 12-lead ECGs lasting between 6 and 60 s (CPSC 2018) | none | dataset standard + 10-fold cv | ResNet | AUC = 0.925 (PTB-XL) AUC = 0.974 (CPSC 2018) |
|  | Śmigiel et al. [275] | 2021 | ECG classification | ECG recordings from the PTB-XL ECG dataset | filtering R wave detection entropy features extraction | train = 70%, val = 15%, test = 15% | CNN | acc = 90.0%, 76.2% and 68.5% for 2, 5 and 20 classes respectively |
|  | Oudkerk Pool et al. [276] | 2021 | R peak detection | 700 randomly chosen ECG recordings from the 2017 PhysioNet/CinC Challenge | manual annotation noise filtering | train = 72%, val = 14%, test = 14% | CNN | pre = 91%, sen = 92.6%, F1 = 91.8% |
|  | Spicher et al. [277] | 2021 | wearable ECG analysis | single-lead ECG recordings from 4 healthy volunteers | segmentation | test = 100% | CNN | latency = 110 ms, data corruption = 0.07% for 5G |
|  | Venton et al. [278] | 2021 | noisy ECG classification | ECG signals from the 2020 PhysioNet/CinC Challenge and the MIT-BIH Noise Stress Test Database | noise filtering noise scaling noise addition SPAR attractors scalograms using CWT | 5-fold cv | AlexNet, GoogLeNet, VGG-16, ResNet-50 | F1 = 70% and 79% using ResNet-50 for SPAR attractors and scalogram transforms respectively |
|  | Mehari and Strodthoff [279] | 2021 | representation learning | ECG recordings from 2020 PhysioNet/CinC Challenge, the Chapman University and Shaoxing People’s Hospital ECG database and the Ribeiro dataset for training  and ECG recordings from the PTB-XL ECG dataset for testing | resampling cropping | 10-fold cv + separate test | ResNet-LSTM | best AUC = 0.9418 |
| ***biometric recognition*** | Jomaa et al. [280] | 2020 | PA detection | 656 ECG recordings from 164 individuals along with fingerprint data | noise filtering segmentation selection | train = 50%, test = 50% | 2D-CNN | acc = 95.32% |
|  | Song et al. [281] | 2020 | person identification | ECG data from the ECG-ID Database and the PTB database, face data from the Faces95 database and fingerprint data from the FVC2006 database  along with ECG, face and fingerprint data from 58 virtual subjects | cropping normalization | 8-fold cv | ResNet-50 | acc = 98.97% for ID classification acc = 96.55% for gender classification |
|  | Belo et al. [282] | 2020 | person identification-authentication | ECG recordings from the Fantasia, MIT-BIH (Arrhythmia, Normal Sinus Rhythm, Long-Term) and CYBHi databases | moving average normalization hanning window convolution edge clipping quantization | train = 33%, test = 67% train = 50%, test = 50% | TCNN | acc = 100%, EER = 0.0% (Fantasia) acc = 96.3%, EER = 0.1% (MIT-BIH) acc = 60.3%, EER = 2.2% (CYBHi) |
|  | AlDuwaile and Islam [283] | 2021 | human recognition | ECG recordings from the PTB Diagnostic ECG Database and the ECG-ID Database | peak detection segmentation entropy enhancement CWT representation | 10-fold cv + 2-fold cv | CNN, GoogLeNet, ResNet, MobileNet, EfficientNet | acc = 99.90% (PTB), 98.20% (ECG-ID mixed-session) and 94.18% (ECG-ID multisession) for the CNN acc = 97.28% (ECG-ID multisession) for the ResNet |
|  | Wu et al. [284] | 2021 | ECG biometric identification | ECG signals from the PTB Diagnostic ECG Database and the CYBHi dataset | baseline wander correction filtering resampling peak detection segmentation | separate train/test | CNN | identification rate > 99% |
|  | Chiu et al. [285] | 2021 | ECG-based biometric recognition | single-lead ECG recordings from the PTB Diagnostic ECG Database | noise filtering segmentation | train = 67%, val = 16.5%, test = 16.5% | CNN | identification rate = 99.1% |
|  | Ghazarian et al. [286] | 2021 | ECG identification | 45,310 12-lead ECG recordings (Chapman, Ningbo) | noise removal RR interval extraction | train = 80%, val = 20% | CNN | acc = 94.56% |
| ***sleep analysis*** | Fonseca et al. [287] | 2020 | sleep staging | PSG recordings of 194 healthy participants, 26 patients with insomnia disorder, 51 patients with sleep apnea, 5 patients with periodic limb movement disorder,  15 patients with Parkinson’s disease and 97 participants who underwent PSG for diagnosis | noise filtering estimation | train = 75%, val = 25% + separate test | BiLSTM | acc = 75.9% |
|  | Sridhar et al. [288] | 2020 | sleep staging | ECG signals from the SHHS, MESA and CinC 2018 | normalization wave detection resampling zero padding | train = 80%, val = 10%, test = 10% | CNN+ResNet | acc = 78% for SHHS acc = 80% for MESA acc = 72% for CinC 2018 |
|  | Chang et al. [289] | 2020 | sleep apnea detection | 34,213 1-min ECG signals from the Apnea-ECG Database | noise filtering standardization | train = 50%, val = 50% | CNN | sen = 81.1%, spe = 92.0%, acc = 87.9% for per-minute apnea detection sen = 95.7%, spe = 100%, acc = 97.1% for per-recording classification |
|  | Sharan et al. [290] | 2020 | sleep apnea detection | 70 overnight ECG recordings | delineation exclusion | train = 50%, test = 50% | 1D-CNN | acc = 88.23%, AUC=0.9453 |
|  | Urtnasan et al. [291] | 2020 | sleep apnea severity identification | 103,690 segments from 144 individuals | segmentation | train = 64%, val = 16%, test = 20% | CNN | F1 = 98.0% |
|  | Jarchi et al. [292] | 2020 | sleep disorder classification | single-lead ECG and EMG recordings from 40 individuals (10 healthy, 10 OSA, 10 RLS and 10 both OSA and RLS) | peak detection resampling | 10-fold cv | DNN | sen = 62%, pre = 53%, acc = 72%, F1 = 57% |
|  | Li et al. [293] | 2021 | cortical arousal during sleep detection | 1,547 single-lead ECG recordings from the Multi-Ethnic Study of Atherosclerosis (MESA) and the Sleep Heart Health Study (SHHS) | standardization | train = 70%, val = 10%, test = 20% (MESA)  train = 55%, val = 5%, test = 40% (SHHS) | CNN+LSTM | AUPRC = 0.62, AUROC = 0.93 |
|  | Mashrur et al. [294] | 2021 | OSA detection | ECG recordings from the Apnea-ECG Database and the St. Vincent's University Hospital / University College Dublin Sleep Apnea Database | noise filtering segmentation removal scalograms using CWT | train = 70%, val = 15%, test = 15% | 2D-CNN | sen = 94.30%, spe = 94.51%, acc = 94.38% (Apnea-ECG) sen = 71.62%, spe = 86.05%, acc = 81.86% (UCDDB) |
|  | Nasifoglu and Erogul [295] | 2021 | OSA detection/prediction | 152 single-lead ECG signals (HomePap and ABC) | baseline wander correction segmentation balancing spectrograms using STFT scalograms using CWT | train = 80%, val = 10%, test = 10% + 10-fold cv | 2D-CNN+ResNet | sen = 86.2%, spe = 85.0%, acc = 85.2% for OSA detection sen = 83.2%, spe = 82.2%, acc = 82.3% for OSA prediction |
|  | Mukherjee et al. [296] | 2021 | sleep apnea detection | ECG recordings from the Apnea-ECG Database | segmentation division peak detection interpolation downsampling standardization | 2-fold cv + 5-fold cv | MLP | acc = 85.58% |
|  | Urtnasan et al. [297] | 2021 | sleep disorders classification | PSG recordings of 35 subjects from the CAP Sleep Database | segmentation | train = 64%, val = 16%, test = 20% | CNN | F1 = 99%, 97%, 97%, 95% and 98% for 5 groups |
|  | Yang et al. [298] | 2021 | OSA detection | ECG recordings from the Apnea-ECG Database and the St. Vincent's University Hospital / University College Dublin Sleep Apnea Database | negated recordings correction overlapping segmentation R-peak detection Q-peak detection padding | train = 50%, test = 50% | CNN | acc = 90.3%, sen = 87.6%, spe = 91.9% |
| ***other clinical analyses*** | Krasteva et al. [299] | 2020 | shock detection | 30-min ECG recordings from 10 patients (AHA fibrillation database), 35-min ECG recordings from 22 patients (VFDB)  and 8-min ECG recordings from 35 patients (CUDB) | exclusion | train = 37%, val = 63% | CNN | sen > 95.2%, spe > 98.6% |
|  | Isasi et al. [300] | 2020 | shock/no-shock decision | 3,319 9s ECG segments (586 shockable and 2,733 non-shockable) | segmentation noise filtering downsampling | 5-fold cv | CNN | sen = 95.8%, spe = 96.1%, acc = 96.1% |
|  | Miura et al. [301] | 2020 | ventilatory threshold estimation | ECG data from 260 cardiovascular patients | conversion to matrix segmentation | train = 37%, val = 28%, test = 35% | 1D-CNN+LSTM | r > 0.7 between DL and ventilatory thresholds for all subgroups |
|  | Kwon et al. [302] | 2020 | PH prediction | 70,709 ECGs from 38,241 patients (4,096 with PH) | noise filtering normalization | train = 90%, val = 10% | CNN+DNN | AUC = 0.859 for internal validation AUC = 0.902 for external validation |
|  | Wang et al. [303] | 2020 | prediabetes diagnosis | 2,251 complete case ECG data as training sets and 663 complete case ECG data as independent test sets | cropping resizing | separate train/test | CNN | acc = 78.1%, AUC = 0.777 |
|  | Ahmad and Khan [304] | 2020 | stress assessment | ECG data from 15 individuals | signal to image conversion resizing DFT GWT | train = 85%, test = 15% | CNN | acc = 85.45% |
|  | Hajeb-M et al. [305] | 2021 | shock decision | 1,131 shockable and 2,741 non-shockable samples contaminated with 43 different CPR artifacts from 40 patients  (MIT-BIH Malignant Ventricular Arrhythmia Database and Sudden Cardiac Death Holter Database) | STFT | 4-fold cv | CNN+ResNet+BiLSTM | sen = 95.21%, spe = 86.03% |
|  | Jekova and Krasteva [306] | 2021 | shock detection | 860 shockable, 3,435 non-shockable and 6,825 asystole rhythms | downsampling SNR estimation | train = 20%, val = 20%, test = 60% | CNN | sen = 89.0%, spe = 91.3% |
|  | Dunn et al. [307] | 2021 | S-ICD implantation eligibility screening | 390 10-second ECG segments (Southampton General Hospital) and 310 20-second ECG segments sampled (ECG-ID Database) | noise filtering peak flipping matrix transformation | 10-fold cv | CNN | MSE = 0.122 |
|  | Kwon et al. [308] | 2021 | electrolyte imbalance detection | ECGs from 92,140 patients (4,638 with electrolyte imbalance) | cropping noise filtering standardization | train = 80%, val = 20% | ResNet | AUC = 0.873, 0.857, 0.839, 0.856, 0.831 and 0.813  for hyperkalemia, hypokalemia, hypernatremia, hyponatremia, hypercalcemia and hypocalcemia |
|  | Ozdemir et al. [309] | 2021 | COVID-19 diagnosis | ECG images from 250 COVID-19 patients, 77 MI patients, 548 patients with abnormal heartbeats (past COVID-19 or MI),  203 patients with past MI and 859 controls | segmentation background noise removal RGB to binary image conversion | 5-fold cv | CNN | acc = 93.00%, F1 = 93.20% |
|  | Noor et al. [310] | 2021 | depression prediction | ECG samples from 5,000 patients | combination | train = 50%, val = 35%, test = 15% | RNN+LSTM | acc = 97.24% for detecting normal heartbeats |
|  | Chang et al. [311] | 2021 | digoxin toxicity detection | 61 ECGs from patients with digoxin toxicity and 177,066 ECGs from patients in the emergency room | exclusion | train = 80%, val = 20% + 5-fold cv | ECG12Net | sen = 84.6%, spe = 96.6% |
|  | Lin et al. [312] | 2021 | DM management | 104,823 ECGs with corresponding HbA1c or fasting glucose, 2,190 ECGs from 1,539 cases for initial validation  and 3,293 cases for DM management | cropping oversampling | train = 95%, val = 2%, test = 3% | CNN+ResNet | sen = 71.9%, spe = 77.7%, AUC = 0.8255 |
|  | Baghersalimi et al. [313] | 2021 | epileptic seizure detection | single-lead ECG and 19-channel EEG data from 30 patients (EPILEPSIAE dataset) | segmentation noise filtering linear detrend standardization | train = 99.999%, val = 0.0001%, test = 0.0001% + LOOCV | 1D-CNN+ResNet | sen = 90.24%, spe = 91.58% |
|  | Russell et al. [314] | 2021 | fatigue prediction | accelerometer and ECG data from one healthy individual | FFT upsampling splitting segmentation | train = 67%, test = 33% | 1D-CNN | MAE = 0.125 - 0.313 for multiple activities |
|  | Bleijendaal et al. [315] | 2021 | PLN mutation diagnosis | ECGs from 155 adult PLN mutation carriers and 155 age- and sex-matched controls | segmentation interpolation | 4-fold cv | 2D-CNN+2D-LSTM | acc = 72%  acc = 67% |
|  | Lopes et al. [316] | 2021 | PLN patient detection | single-ECGs from a total of 256,278 patients for training and ECG recordings from the PLN dataset (155 with PLN) for finetuning | exclusion resampling | 10-fold cv | CNN | AUROC = 0.87 using transfer learning and AUROC = 0.71 with training from scratch (balanced population) AUROC = 0.90 using transfer learning and AUROC = 0.65 with training from scratch (imbalanced population) |
|  | Lin et al. [317] | 2021 | TPP diagnosis | 50 ECGs from 42 TPP patients, 502 ECGs from 414 hypokalemic controls and 36 ECGs from 36 non-TPP controls | cropping oversampling | 5-fold cv | ECG12Net | MAE = 0.26 mEq/L for hypokalemia detection in TPP patients AUC = 0.8 for TPP diagnosis |
|  | Mazumder et al. [318] | 2021 | shockable rhythm detection | ECG recordings from the MIT-BIH Malignant Ventricular Arrhythmia Database and the Creighton University Ventricular Tachycardia Database | mean subtraction filtering exclusion windowing | 5-fold cv | CNN-LSTM | sen = 96.10%, spe = 98.34% (CUDB 2-s window) sen = 94.68%, spe = 92.77% (VFDB 2-s window) sen = 99.21%, spe = 99.68% (CUDB 8-s window) sen = 98.56%, spe = 99.08% (VFDB 8-s window) |
|  | He et al. [319] | 2021 | ECG denoising | ECG signals from the MIT-BIH Arrhythmia Database and the MIT-BIH Noise Stress Test Database | segmentation noise addition normalization | train = 80%, val = 10%, test = 10% | AE-CNN | 8dB improvement under -4 dB noise |
|  | Li et al. [320] | 2021 | emotion recognition | 3-lead ECG signals from 16 subjects | filtering segmentation peak detection | LOOCV | CNN-LSTM | acc = 77.41% for three classes |
|  | Kwon et al. [321] | 2021 | sepsis screening | 10s 12-lead ECG signals from 46,017 patients | cropping standardization noise filtering normalization noise addition | train = 40%, val = 17%, test = 43% | ResNet | AUC = 0.863 for screening sepsis AUC = 0.899 for detecting septic shock |
|  | Sarkar et al. [322] | 2021 | maternal and fetal stress detection | fECG and mECG recordings from 107 participants | resampling segmentation exclusion | 5-fold cv | CNN | AUROC = 0.982 |
